# Longitudinal patterns of the relation between anxiety, depression and posttraumatic stress disorder among postpartum women with and without maternal morbidities in Northwest Ethiopia: a cross-lagged autoregressive structural equation modelling

**DOI:** 10.1186/s13690-022-00978-0

**Published:** 2022-10-29

**Authors:** Marelign Tilahun Malaju, Getu Degu Alene

**Affiliations:** 1 Department of Public Health, College of Health Sciences, Debre Tabor University, Debre Tabor, Ethiopia; 2 School of Public Health, College of Medicine and Health Sciences, Bahir Dar University, Bahir Dar, Ethiopia

**Keywords:** Anxiety, Depression, Posttraumatic stress disorder, Cross-lagged, Autoregressive, Structural equation modelling, Maternal morbidities, Postpartum women

## Abstract

**Background:**

The postpartum period is a time where mothers can undergo significant changes that increase vulnerability for depression, anxiety and posttraumatic stress disorder symptoms.

However, the direct and indirect factors of depression, anxiety and posttraumatic stress disorder symptoms and their direction of relationships following childbirth is not well investigated in Ethiopia. The aim of this study was to determine the direct and indirect factors of depression, anxiety and posttraumatic stress disorder symptoms and their direction of relationships following childbirth.

**Methods:**

A total of 775 women consented to participate at the first, second and third follow-up of the study (6^th^, 12^th^ and 18^th^ week of postpartum period) during October, 2020 – March, 2021.

Women were recruited after childbirth and before discharge using the World Health Organization maternal morbidity working group criteria to identify exposed and non-exposed groups. A cross-lagged autoregressive path analysis and linear structural equation modelling were carried out using Stata version 16 software.

**Results:**

Prevalence rates of anxiety were 18.5%, 15.5% and 8.5% at the 6^th^, 12^th^ and 18^th^ week of postpartum respectively. The prevalence rates for depression were also found to be 15.5%, 12.9% and 8.6% respectively during the same follow up period and for posttraumatic stress disorder it was found to be 9.7%, 6.8% and 3.5% at the 6^th^, 12^th^ and 18^th^ week of postpartum respectively. Moreover, anxiety and depression were found to be a causal risk factors for posttraumatic stress disorder in the postpartum period. Direct maternal morbidity, fear of childbirth, higher gravidity, perceived traumatic childbirth and indirect maternal morbidity were found to have a direct and indirect positive association with depression, anxiety and posttraumatic stress disorder. In contrast, higher parity, higher family size and higher social support have a direct and indirect negative association.

**Conclusion:**

Postnatal mental health screening, early diagnosis and treatment of maternal morbidities, developing encouraging strategies for social support and providing adequate information about birth procedures and response to mothers’ needs during childbirth are essential to improve maternal mental health in the postpartum period.

**Supplementary Information:**

The online version contains supplementary material available at 10.1186/s13690-022-00978-0.

## Background

The  postpartum period is a time where mothers can undergo significant changes that increase vulnerability for depression, anxiety and posttraumatic stress disorder symptoms [1, 2]. Depression is a state of low mood or loss of pleasure or interest in activities, while anxiety is generally characterized by feelings of tension, worried thoughts and physical changes [3]. Post-traumatic stress disorder (PTSD) refers to a cluster of psychological symptoms that develop following exposure to a severe stressor or traumatic event associated with a real or perceived threat of death or threat to physical integrity of the person or others. Symptom clusters involve re-experiencing the event (intrusion symptoms), persistent avoidance of stimuli associated with the event (avoidance symptoms), negative mood alterations, and increased arousal and reactivity [3].

The prevalence rates of postnatal depression in low- and middle-income countries (LMICs) ranged between 10 to 20% [4]. For anxiety, the global estimates are about 8–10% during the postnatal period [5]. In a systematic review, it has been reported that rates of depression were 18.3% during the postnatal periods, whereas prevalence rates of postnatal anxiety were 14% [6]. Other studies, however, have found even higher rates. A study conducted in Cape Town, found that 34.7% of postnatal women with depression symptoms [7]. In a study conducted in Uganda, 43% of the participants were found to have postpartum depressive symptoms [8]. A systematic review reported that the prevalence of anxiety disorder is 17.1% after childbirth [9]. Another meta-analysis showed a postpartum period prevalence of 13.7% for anxiety symptoms [10] In a review of studies from Africa, the postnatal anxiety prevalence rate was 14% [6].

There is also increasing evidence that women may perceive childbirth as traumatic and develop PTSD as a result of a traumatic birth [11–13]. Traumatic birth has been defined as an event occurring during labour and birth that may be a serious threat to the life and safety of the mother and/or child [14]. It has been reported that 9 to 45.5% of mothers perceived childbirth as traumatic [11, 15, 16] and estimates of post-traumatic stress disorder in the postpartum period were reported to be 3–15% in previous literatures [17–20]. A meta-analysis reported that 4% of women were found to have PTSD symptoms in the postpartum period and this increases to 18.5% in women with complications in pregnancy [20]. A large study of women in Norway reported that 1.8% of women had severe PTSD following childbirth [21], whereas in Iran it has been found that 20% of women had severe PTSD following childbirth [22].

Postpartum PTSD is the outcome of the interplay between antepartum vulnerability factors, the events during delivery and postpartum factors that interact over time during perinatal period [18]. PTSD following childbirth usually occurs as a result of complications during pregnancy or birth [17, 23]. However, it may also be a continuation of pre-existing PTSD, a reactivation of PTSD triggered by childbirth related events that had previously resolved or new-onset PTSD in response to an event which is not related to childbirth [12, 24, 25].

Three patterns may account for the direction of relationships between symptoms of depression, anxiety and PTSD. First preexisting psychiatric disorders increase the risk for PTSD, either by increasing the risk for exposure to traumatic events or increase victims' susceptibility to the PTSD inducing effects of trauma. In this regard, the onset of depression and anxiety precedes PTSD onset and may increase the risk for PTSD (i.e., the affective dysregulation model). Second, PTSD leads to anxiety and depression via its demoralizing effect, which thus may be considered as complications of the PTSD and its impairment (the demoralization model). Third, PTSD, anxiety and depression are independent disorders which co-occur due to shared risk factors. Symptoms of depression, anxiety and PTSD all emanating from a common source; hence they are synchronous (i.e., the synchronous model) [26–28].

However, the direction of relationships between symptoms of depression, anxiety and PTSD following childbirth is not well investigated and there is no study conducted in Ethiopia in this regard. Thus, if symptoms of depression, anxiety and PTSD are synchronous following delivery, then preventive intervention efforts should be geared towards eliminating all of them. If, however, one type of symptoms leads to the other, then the leading type must be treated first, possibly preventing the second and third. Therefore, the aim of this study was to determine the direct and indirect factors of depression, anxiety and PTSD symptoms and their direction of relationships following childbirth.

## Methods

### Study design and study area

This study was part of the health facility linked community based prospective follow-up study conducted in Northwest Ethiopia to determine the effect of maternal morbidities on maternal health related quality of life, functional status and mental health problems [29–31]. Postpartum women were recruited in four hospitals of south Gondar zone, Northwest Ethiopia. The data collection took place between October 1, 2020 and March 30, 2021. South Gondar is located at 650 km Northwest from Addis Ababa the capital city of Ethiopia.

### Study population

A total of 775 women consented to participate in the study and participated at the first, second and third follow-up of the study (6^th^, 12^th^ and 18^th^ week of postpartum period). Recruitment of the study participants was done after child birth and before the time of discharge. Women with any of the direct maternal morbidities were recruited into the exposed group and those without the direct maternal morbidities were into the non-exposed group based on the WHO maternal morbidity criteria [32].

### Sample size determination

The total sample size was determined by using Epi-Info version 7 with a two-population proportion formula. Hence, a sample size of 779 was obtained by taking 0.05 alpha (α), power of 90%, odds ratio of 4.23, proportion of 2.3%, ratio of 1:3 and by adding 15% non-response rate. These sample size calculation values were obtained from a previous study [24].

### Eligibility/inclusion criteria

Women aged 15 years and above, with preterm, term or post term delivery and with live birth, still birth or fetal death were included in the study. The PTSD criterion A was not considered as an exclusion criterion, because childbirth related negative events and emotions that do not satisfy the criterion A can cause symptoms that could qualify as a PTSD diagnosis [13].

### Sampling procedure

**Fig. 1:**
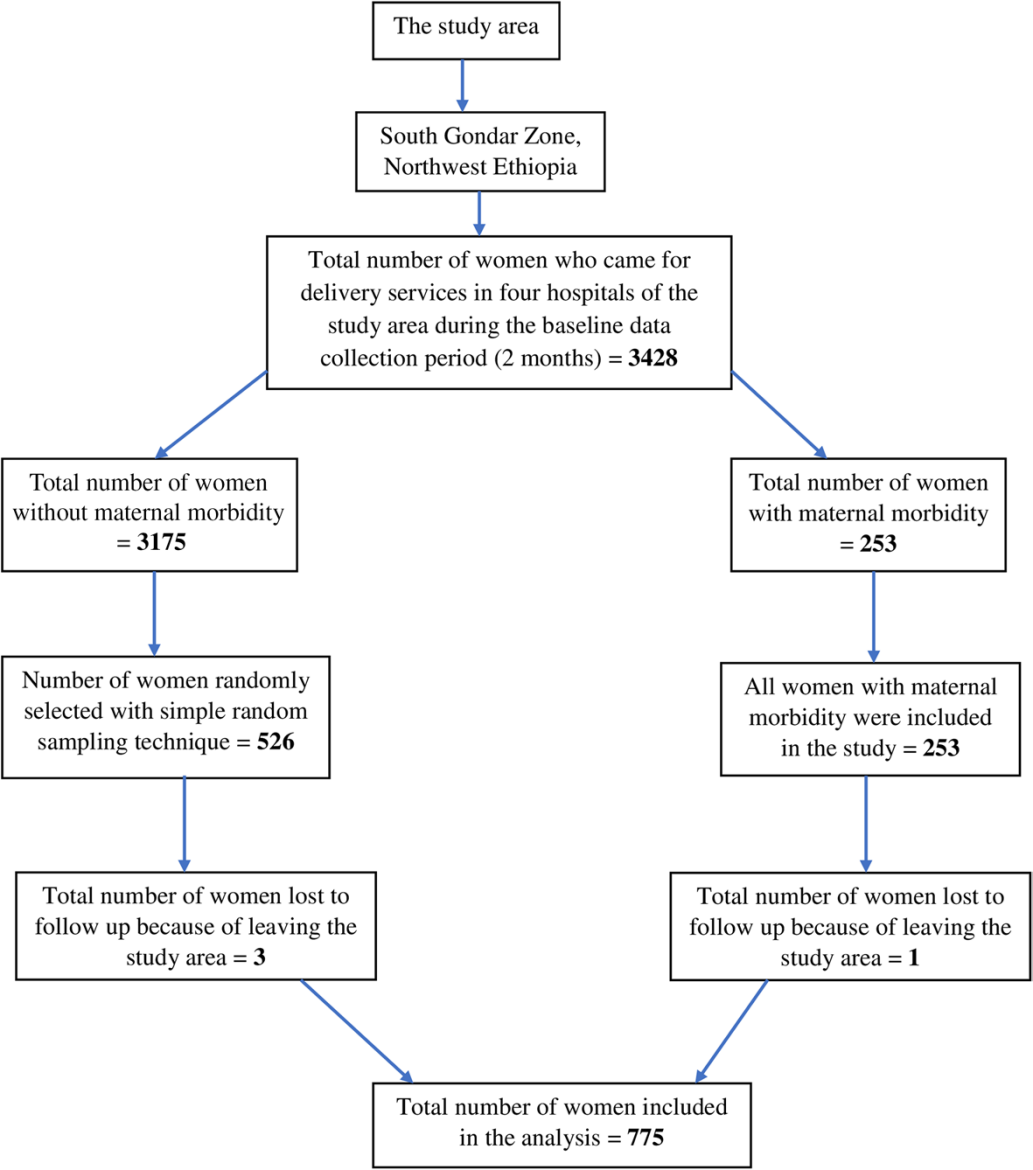
A flow diagram chart of study participants sampling procedure

All exposed women with direct maternal morbidity included in the study and non-exposed women without direct maternal morbidities were selected by simple random sampling method using their chart number on daily bases. With 1:2 ratio of exposed to non-exposed mothers, this recruitment procedure continued prospectively until the required sample size was fulfilled. Women were asked for consent to participate in the study and after getting their consent and full address, appointments were made at their home to collect the data for the follow up study. The study participants overall sampling procedure is shown in Fig. 1.

### Outcome and independent variables

The outcome variables were depression, anxiety and posttraumatic stress disorder. The independent variables were; direct maternal morbidities(obstetric hemorrhage, hypertensive disorders, obstructed labour, puerperal sepsis, gestational diabetes mellitus, perineal tear), indirect maternal morbidities (anemia, malaria, hypertension, asthma, tuberculosis, HIV), socio-demographic variables (age, educational status, marital status, religion, ethnicity, occupation, monthly expenditure), obstetric variables (parity, mode of delivery, gestational age at birth, birth weight, birth interval, fetal death, unwanted pregnancies, antenatal care visit, history of abortion), residence and psychosocial factors (social support and fear of child birth).

### Measures of variables

#### Depression, anxiety and stress

The short version of depression, anxiety and stress scale 21 (DASS-21) questionnaire was used to measure depression, anxiety and stress. The instrument has 21 items with three domains. Each domain comprises seven items assessing symptoms of depression, anxiety and stress. In this study a score ≥ 10 was considered for a mother to have a symptom of depression. A cut-off score of ≥ 8 was considered to have symptoms of anxiety and a score of ≥ 15 was considered to have symptoms of stress. This instrument was used previously in Ethiopia [33, 34].

### Posttraumatic stress disorder

The childbirth stressor was operationalized by using the Traumatic Event Scale (TES) [35, 36]. In this scale, the items concerning criterion A (stressor) were formulated as follows:

- 1. “The childbirth was a trying experience.”
- 2. “The childbirth was a threat to my physical integrity.”
- 3. “During the childbirth I was afraid that I was going to die.”
- 4. “During the childbirth I felt anxious/helpless/horrified.”

Four alternative answers follow each statement: “not at all,” “somehow,” “much,” and “very much.” Criterion A is fulfilled if either of the alternatives “much” or “very much” on item 1, 2 and/or 3, and 4 is marked [35, 36].

After the questions regarding criterion A, we have used the Posttraumatic Stress Disorder Checklist for DSM-5 (PCL-5) comprising the 20 PTSD symptoms (criterion B, C, D and E) to measure PTSD. The instrument contains 20 items, including three new PTSD symptoms (compared with the PTSD Checklist for DSM-V): blame, negative emotions and reckless or self-destructive behavior [37]. A total-symptom score of zero to 80 can be obtained by summing the items. A score of 31–33 is optimal to determine PTSD symptoms and a score of ≥ 33 is recommended when further psychometric testing is not available [38, 39]. Therefore, a score of ≥ 33 was considered to have symptoms of PTSD for this study. The instrument was used previously in Ethiopia [39].

### Fear of child birth

The Wijma Delivery Expectation/Experience Questionnaire (W-DEQ) was used to measure fear of child birth. The W-DEQ has been designed specially to measure fear of child birth operationalized by the cognitive appraisal of the delivery. This 33-item rating scale has a 6-point Likert scale as a response format, ranging from ' not at all' (= 0) to ' extremely' (= 5), yielding a score-range between 0 and 165. The Internal consistency and split-half reliability of the W-DEQ was checked in previous studies in Ethiopia with the Cronbach's alpha score of 0.932 [40, 41]. A score of ≥ 85 was considered to have fear of child birth for this study [40, 41].

### Social support

The Oslo 3-items social support scale with scores ranging from 3 to 14 was used to measure social support. The social support scores were categorized into poor or no social support for scores less than nine. Scores between 9 and 14 were considered moderate to strong support and merged together as “yes” for social support. The Oslo 3-items social support scale was validated and previously used in Ethiopia [42–44].

### Data collection and quality control

Administering baseline questionnaire and diagnosis of direct and indirect maternal morbidities based on the WHO criteria, were done by health professionals working in the Gynecology and Obstetrics wards of the study Hospitals. The questionnaire consisted of a patient interview and record review. The interview was on socioeconomic status, medical and obstetric history and clinical symptoms. The record review was intended to extract information on selected laboratory tests and results for hemoglobin, HIV, malaria (rapid diagnostic test or smear) and glucometer (random blood sugar). The DASS-21 and PCL-5 were administered by health extension workers at the first, second and third home visit (6^th^, 12^th^ and 18^th^ week of postpartum period). Training was given for data collectors and supervision was done by the principal investigator. During the training process, data collectors carefully reviewed each question and conduct pretest before the study commences. The investigator and data collectors have checked the final version of the questionnaire and update as required based on the pretest.

### Data processing and analysis

A three-wave cross-lagged autoregressive structural equation modeling was carried out using Stata version 16 software [45]. The Autoregressive Cross-lagged (ARCL) modeling strategy was used to examine the longitudinal relations between PTSD, depression, and anxiety [26, 45, 46]. This modeling strategy incorporates three main components. First, the stability/autoregressive effects (effects of T1 depressive, anxiety and PTSD symptoms on their respective T2 variables). That means, later measures of a construct are predicted by earlier measures of the same construct. Second, the cross-lagged effects (effect of T1 depressive symptoms on T2 PTSD symptoms and of T1 PTSD symptoms on T2 depressive symptoms). That means, earlier measures of depression predict later measures of PTSD. This model can be extended to examine bi-directional relations such that earlier measures of PTSD predict later measures of depression as well. Third, the synchronous associations between the unexplained variances of these variables at T1, T2 and T3 [26, 46].

We estimated the model fitness by using the comparative fit index (CFI), Tucker-Lewis Index (TLI) and the root-mean-square error of approximation (RMSEA). Both the TLI and CFI should be greater than 0.90, but the RMSEA value should be less than 0.08 to judge the model as reasonably fitting the data [13, 46]. The aim of the analysis was to examine the cross-lagged effects of depressive, anxiety and PTSD symptoms, controlling for the confounder variables and stability/autoregressive effects. In addition, the direct and indirect relationships between the independent and dependent variables was also explored using the structural equation modeling. This allowed us to assess the strength of the hypothesized direct and indirect causal pathways. Estimated effects for which *p* < 0.05 were considered as being statistically significant.

### Ethical considerations

This study was approved by the institutional review board of Bahir Dar University. Each study participant has given written informed consent before participating in the study. Assent was also obtained from teenage mothers whose age is less than 18 years, in addition to informed consent from their care givers. Using codes, passwords and limiting access to the data only for the investigators were the measures taken to ensure the confidentiality of the data. Data collectors read out and assisted participants to fill out the consent form if participants were unable to read and write.

## Results

The total number of women recruited at baseline were 779 and out of this, 775(99.5%) of them participated at the first, second and third follow-up of the study (6^th^, 12^th^ and 18^th^ week of postpartum period). Four mothers were lost to follow up because of changing their place of living and going out of the study area. The mean age of the study participants was 26.3(4.36). Almost all of them 774(99.9%) were Amhara by ethnicity and 742(95.7%) were followers of Orthodox Christianity. Other socio-demographic characteristics of mothers are shown in Table 1.

**Table 1:** Socio-demographic characteristics of postpartum women in Northwest Ethiopia, 2021

Variables	Direct maternal morbidity		Total *n* (%)
	Yes. *n* (%)	No. *n* (%)	
**Age** [Mean(± SD) = 26.33(± 4.355)]			
**Residence**			
Urban	251 (32.4)	520 (67.1)	771(99.5)
Rural	1 (0.1)	3 (0.4)	4(0.5)
**Ethnicity** Amhara Tigre			
Amhara	252 (32.5)	522 (67.4)	774 (99.9)
Tigre	0 (0.0)	1 (0.1)	1 (0.1)
**Religion** Orthodox Muslim Protestant			
Orthodox	241 (31.1)	501 (64.6)	742 (95.7)
Muslim	10 (1.3)	20 (2.6)	742 (95.7)
Protestant	1 (0.1)	2 (0.3)	3 (0.4
**Education status**			
Illiterate/read and write	31 (4.0)	34 (4.4)	65 (8.4)
Grade 1–8	48 (6.2)	88 (11.4)	136 (17.5)
Grade 9–12	74 (9.5)	145 (18.7)	219 (28.3)
Certificate/Diploma	63 (8.1)	154 (19.9)	217 (28.0)
Degree and higher	36 (4.6)	102 (13.2)	138 (17.8)
**Occupation**			
Gov't employed	61 (7.9)	169 (21.8)	230 (29.7)
Merchant/Student	39 (5.0)	106 (13.7)	145 (18.7)
Housewife	141 (18.2)	226 (29.2)	367 (47.4)
Farmer/Daily laborer	11 (1.4)	22 (2.8)	33 (4.3)
**Marital Status**			
Married	246 (31.7)	516 (66.6)	762 (98.3)
Single/widowed/divorced	6 (0.8)	7 (0.9)	13 (1.7)
**Monthly expenditure**			
< = 3000 Ethiopian currency	48 (6.2)	158 (20.4)	206 (26.6)
3001–4000 Ethiopian currency	76 (9.8)	116 (15.0)	192 (24.8)
> = 4001 Ethiopian currency	128 (16.5)	249 (32.1)	377 (48.6)

### Prevalence rates of depression, anxiety and PTSD symptoms by direct maternal morbidity status in the postpartum period

The prevalence of depression, anxiety and PTSD symptoms at the 6^th^, 12^th^ and 18^th^ week of postpartum period was computed and is provided in Table 2. The most common disorder was anxiety followed by depression throughout the follow up period. PTSD symptom was rarely reported at each time point.

**Table 2:** Prevalence of depression, anxiety and PTSD symptoms by direct maternal morbidity status among postpartum women in Northwest Ethiopia, 2021

Postpartum Follow up time			Type of mental health disorder by direct maternal morbidity status						Perceived traumatic childbirth	
			**PTSD symptom**		**Depression**		**Anxiety**			
			**Yes** **n (%)**	**No** **n (%)**	**Yes** **n (%)**	**No** **n (%)**	**Yes** **n (%)**	**No** **n (%)**	**Yes** **n (%)**	**No** **n (%)**
**6** ^**th**^ ** week**	**Direct maternal** **morbidity**	**Yes**	11(1.4)	241(31.1)	24(3.1)	228(29.4)	33(4.3)	219(28.3)	111(14.3)	141(18.2)
		**No**	64(8.3)	459(59.2)	96(12.4)	427(55.1)	110(14.2)	413(53.3)	195(25.2)	328(42.3)
**Total**			75(9.7)	700(90.3)	120(15.5)	655(84.5)	143(18.5)	632(81.5)	306(39.5)	469(60.5)
**P-value**			**0.001**		**0.001**		**0.008**		**0.071**	
**12** ^**th**^ ** week**	**Direct maternal** **morbidity**	**Yes**	8(1.0)	244(31.5)	10(1.3)	242(31.2)	16(2.1)	236(30.5)		
		**No**	45(5.8)	478(61.7)	90(11.6)	433(55.9)	104(13.4)	419(54.1)		
**Total**			53(6.8)	722(93.2)	100(12.9)	675(87.1)	120(15.5)	655(84.5)		
**P-value**			**0.005**		** < 0.001**		** < 0.001**			
**18** ^**th**^ ** week**	**Direct maternal** **morbidity**	**Yes**	5(0.6)	247(31.9)	8(1.0)	244(31.5)	11(1.4)	241(31.1)		
		**No**	22(2.8)	501(64.6)	59(7.6)	464(59.9)	63(8.1)	460(59.4)		
**Total**			27(3.5)	748(96.5)	67(8.6)	708(91.4)	74(9.5)	701(90.5)		
**P-value**			**0.114**		** < 0.001**		**0.001**			
**Total number of women**			**775**		**775**		**775**		**775**	

### Risk trends of developing depression, anxiety and PTSD symptoms over the follow up period

The results of adjusted autoregressive models in Table 3 showed that the risk of suffering from depression, anxiety and PTSD symptoms decreases over time. The risk of suffering from PTSD symptoms at time 2 for women having PTSD symptoms at time 1 was 0.90 (95% CI = 0.88, 0.93). For women who had PTSD symptoms at time 2, this risk dropped to 0.83 (95% CI = 0.79, 0.87) at time 3. Similar to this, for women who had depression at time 1, the chance of developing it at time 2 was 0.78 (95% CI = 0.73, 0.82) and it decreased to 0.74 (95% CI = 0.67, 0.80) at time 3 for those who had depression at time 2. The risk of developing anxiety symptoms at time 2 was 0.88 times for women who had anxiety at time 1 and this risk had reduced to 0.82 at time 3 for those women with anxiety at time 2.

**Table 3:** Trends of the risks for developing depression, anxiety and PTSD symptoms over the follow up period, among postpartum women in Northwest Ethiopia, 2021

Type of mental health disorder symptoms	Trends of the risks for developing each mental health disorder symptoms over the follow up period					
	**From Time 1 to Time 2**			**From Time 2 to Time 3**		
	**Standardized β (95%CI)**	**SE**	***P*** **-value**	**Standardized β (95%CI)**	**SE**	***P*** **-value**
PTSD symptoms	0.90(0.88, 0.93)	0.01	< 0.001	0.83(0.79, 0.87)	0.02	< 0.001
Depression symptoms	0.78(0.73, 0.82)	0.02	< 0.001	0.74(0.67, 0.80)	0.03	< 0.001
Anxiety symptoms	0.88(0.84, 0.93)	0.02	< 0.001	0.82(0.76, 0.89)	0.03	< 0.001

### The longitudinal direction of association between depression, anxiety and PTSD symptoms without controlling the direct and indirect effects of confounding variables

In order to investigate the longitudinal relations between PTSD, depression and anxiety, a cross-lagged autoregressive structural equation modeling was carried out.

### Direction of association between anxiety and PTSD without controlling the direct and indirect effects of confounding variables

The results of a cross-lagged autoregressive structural equation modelling for the theoretical/hypothetical model can be found in the online Supplemental files 1, 2, 3, 4 and 5. We have also provided the software functions used for data analysis in the online Supplemental files 6, 7, 8, 9, 10, 11, 12, 13, 14, 15, 16 and 17.

**Fig. 2:**
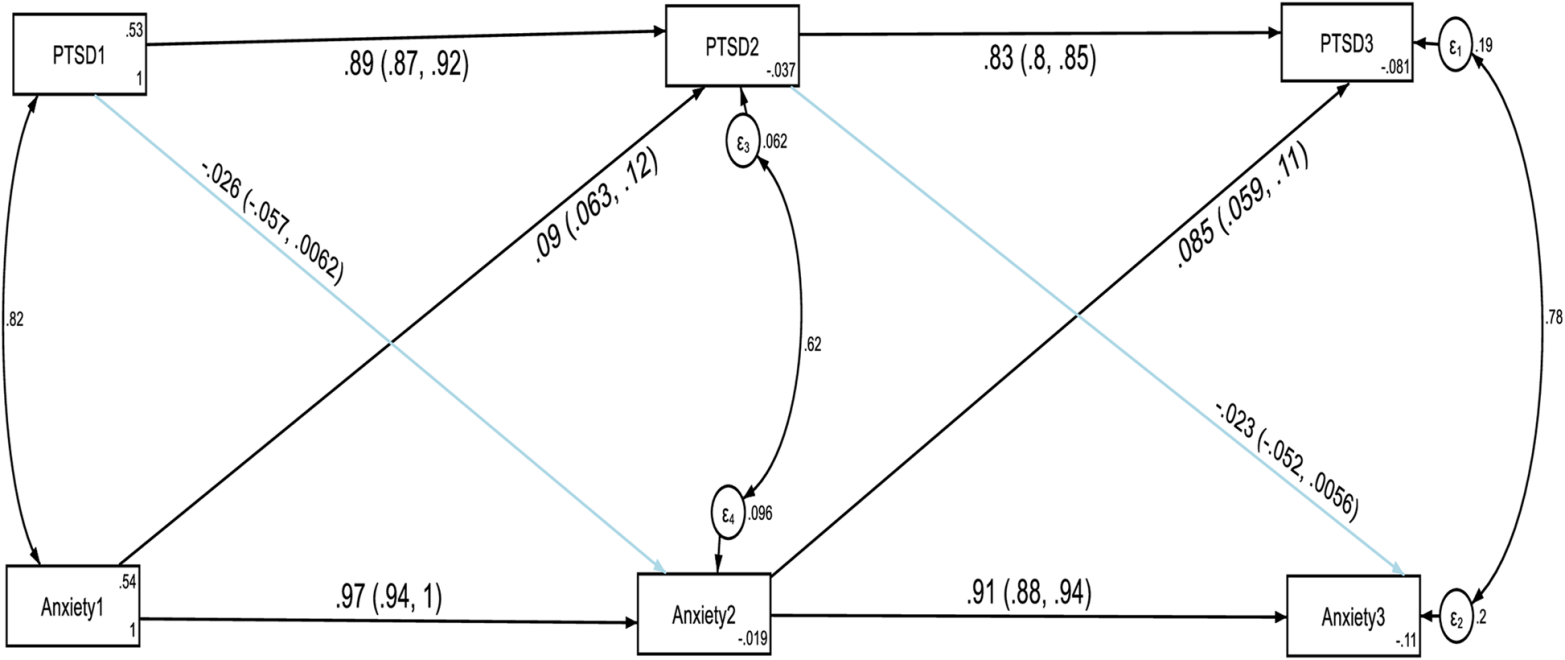
Cross-lagged autoregressive model assessing longitudinal stability and cross-lagged effects between anxiety and PTSD without controlling the direct and indirect effects of confounding variables. *Note:* All β’s are standardized estimates with their 95% CI

The temporal invariance of the structural model for anxiety and PTSD across three data points in time was tested by conducting a constrained and unconstrained cross-lagged autoregressive analysis. A constrained model where the autoregressive and cross-lagged path coefficients were constrained to be equal across three time points did not significantly differ from the unconstrained model where the parameters were freely estimated as evidenced by the fit indices test of difference (ΔCFI = 0.006, ΔTLI = 0.01, ΔRMSEA = 0.048). Model fit for the unconstrained model: CFI = 0.999, TLI = 0.998, SRMR = 0.002 and RMSEA = 0.042. Model fit for the constrained model: CFI = 0.993, TLI = 0.988, SRMR = 0.056 and RMSEA = 0.09. Thus, these results indicated that anxiety and PTSD showed factorial invariance across the three waves and we used the constrained model as the final approved model of this study. The results of the structural model are given in Fig. 2 and Table 4.

**Table 4:** Results of the autoregressive analysis between depression, anxiety, PTSD symptoms without controlling the direct and indirect effects of confounding variables among postpartum women in Northwest Ethiopia, 2021

**The autoregressive analysis between anxiety and PTSD**			
**The Autoregressive effect results**	**Standardized β (95%CI)**	**SE**	***P*** **-value**
**Prediction of:**			
T2 Anxiety by T1 Anxiety symptoms	0.97(0.94, 0.99)	0.014	< 0.001
T3 Anxiety by T2 Anxiety symptoms	0.91(0.88, 0.94)	0.014	< 0.001
T2 PTSD by T1 PTSD symptoms	0.89(0.87, 0.92)	0.012	< 0.001
T3 PTSD by T2 PTSD symptoms	0.83(0.80, 0.85)	0.013	< 0.001
**The autoregressive analysis between depression and PTSD**			
**The Autoregressive effect results**	**Standardized β (95%CI)**	**SE**	***P*** **-value**
**Prediction of:**			
T2 Depression by T1 Depression symptoms	0.96(0.93, 0.98)	0.014	< 0.001
T3 Depression by T2 Depression symptoms	0.90(0.87, 0.93)	0.014	< 0.001
T2 PTSD by T1 PTSD symptoms	0.89(0.87, 0.92)	0.012	< 0.001
T3 PTSD by T2 PTSD symptoms	0.83(0.80, 0.85)	0.013	< 0.001
**The autoregressive analysis between depression and anxiety**			
**The Autoregressive effect results**	**Standardized β (95%CI)**	**SE**	***P*** **-value**
**Prediction of:**			
T2 Depression by T1 Depression symptoms	0.79(0.74, 0.84)	0.025	< 0.001
T3 Depression by T2 Depression symptoms	0.75(0.70, 0.79)	0.024	< 0.001
T2 Anxiety by T1 Anxiety symptoms	0.86(0.81, 0.91)	0.027	< 0.001
T3 Anxiety by T2 Anxiety symptoms	0.81(0.75, 0.86)	0.026	< 0.001
**The Cross-lagged effect results**	**Standardized β (95%CI)**	**SE**	***P*** **-value**
**Prediction of:**			
T2 PTSD by T1 anxiety symptoms	0.09(0.06, 0.12)	0.014	< 0.001
T3 PTSD by T2 anxiety symptoms	0.085(0.06, 0.11)	0.013	< 0.001
T2 PTSD by T1 depression symptoms	0.088(0.06, 0.12)	0.015	< 0.001
T3 PTSD by T2 depression symptoms	0.084(0.06, 0.11)	0.014	< 0.001
T2 anxiety by T1 depression symptoms	0.097(0.04, 0.15)	0.03	< 0.001
T3 anxiety by T2 depression symptoms	0.09(0.04, 0.14)	0.03	< 0.001
T2 depression by T1 anxiety symptoms	0.17(0.12, 0.22)	0.03	< 0.001
T3 depression by T2 anxiety symptoms	0.16(0.11, 0.21)	0.02	< 0.001

The autoregressive path coefficients revealed that anxiety at T1 and PTSD at T1 predicted anxiety at T2 and PTSD at T2 respectively without controlling the direct and indirect effects of confounding variables. In addition, anxiety at T2 and PTSD at T2 predicted anxiety at T3 and PTSD at T3 respectively without controlling the direct and indirect effects of confounding variables. The cross-lagged path coefficients showed that anxiety at T1 significantly predicted PTSD at T2 (β = 0.09, *p*-value < 0.001) without controlling the direct and indirect effects of confounding variables. Moreover, anxiety at T2 significantly predicted PTSD at T3 (β = 0.085, *p*-value < 0.001). However, PTSD did not significantly predict anxiety at different time points. In other words, suffering from anxiety at T1 increased the likelihood of PTSD at T2 and suffering from anxiety at T2 increased the likelihood of PTSD at T3. However, suffering from PTSD did not change the likelihood of anxiety in subsequent waves of measurement without controlling the direct and indirect effects of confounding variables.

### Direction of association between depression and PTSD symptoms without controlling the direct and indirect effects of confounding variables

**Fig. 3:**
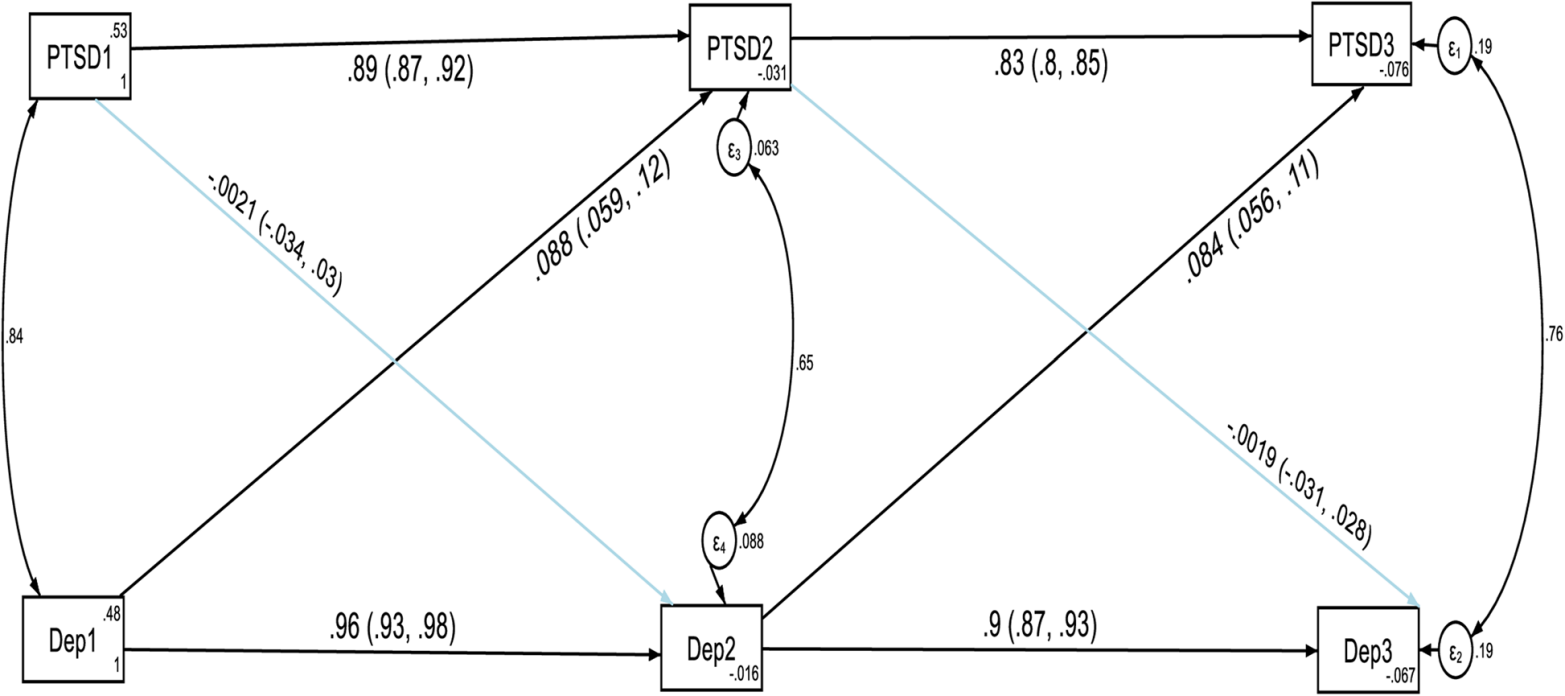
Cross-lagged autoregressive model assessing longitudinal stability and cross-lagged effects between depression and PTSD without controlling the direct and indirect effects of confounding variables. *Note:* All β’s are standardized estimates with their 95% CI

As we did for anxiety and PTSD, the constrained model for depression and PTSD where the autoregressive and cross-lagged path coefficients were constrained to be equal across three time points did not significantly differ from the unconstrained model where the parameters were freely estimated as evidenced by the fit indices test of difference (ΔCFI = 0.005, ΔTLI = 0.007, ΔRMSEA = 0.03). Model fit for the unconstrained model: CFI = 0.998, TLI = 0.995, SRMR = 0.002 and RMSEA = 0.062. Model fit for the constrained model: CFI = 0.993, TLI = 0.988, SRMR = 0.044 and RMSEA = 0.092. Thus, these results indicated that depression and PTSD showed factorial invariance across the three waves and we used the constrained model as the final approved model of this study. The results of the structural model are given in Fig. 3 and Table 4.

The autoregressive path coefficients revealed that depression at T1 and PTSD at T1 predicted depression at T2 and PTSD at T2 respectively without controlling the direct and indirect effects of confounding variables. In addition, depression at T2 and PTSD at T2 predicted depression at T3 and PTSD at T3 respectively without controlling the direct and indirect effects of confounding variables. The cross-lagged path coefficients showed that depression at T1 significantly predicted PTSD at T2 (β = 0.088, *p*-value < 0.001) without controlling the direct and indirect effects of confounding variables. Moreover, depression at T2 significantly predicted PTSD at T3 (β = 0.0845, *p*-value < 0.001) without controlling the direct and indirect effects of confounding variables. However, PTSD did not significantly predict depression at different time points. In other words, suffering from depression at T1 increased the likelihood of PTSD at T2 and suffering from depression at T2 increased the likelihood of PTSD at T3. However, suffering from PTSD did not change the likelihood of depression in subsequent waves of measurement without controlling the direct and indirect effects of confounding variables.

### Direction of association between depression and anxiety without controlling the direct and indirect effects of confounding variables

**Fig. 4:**
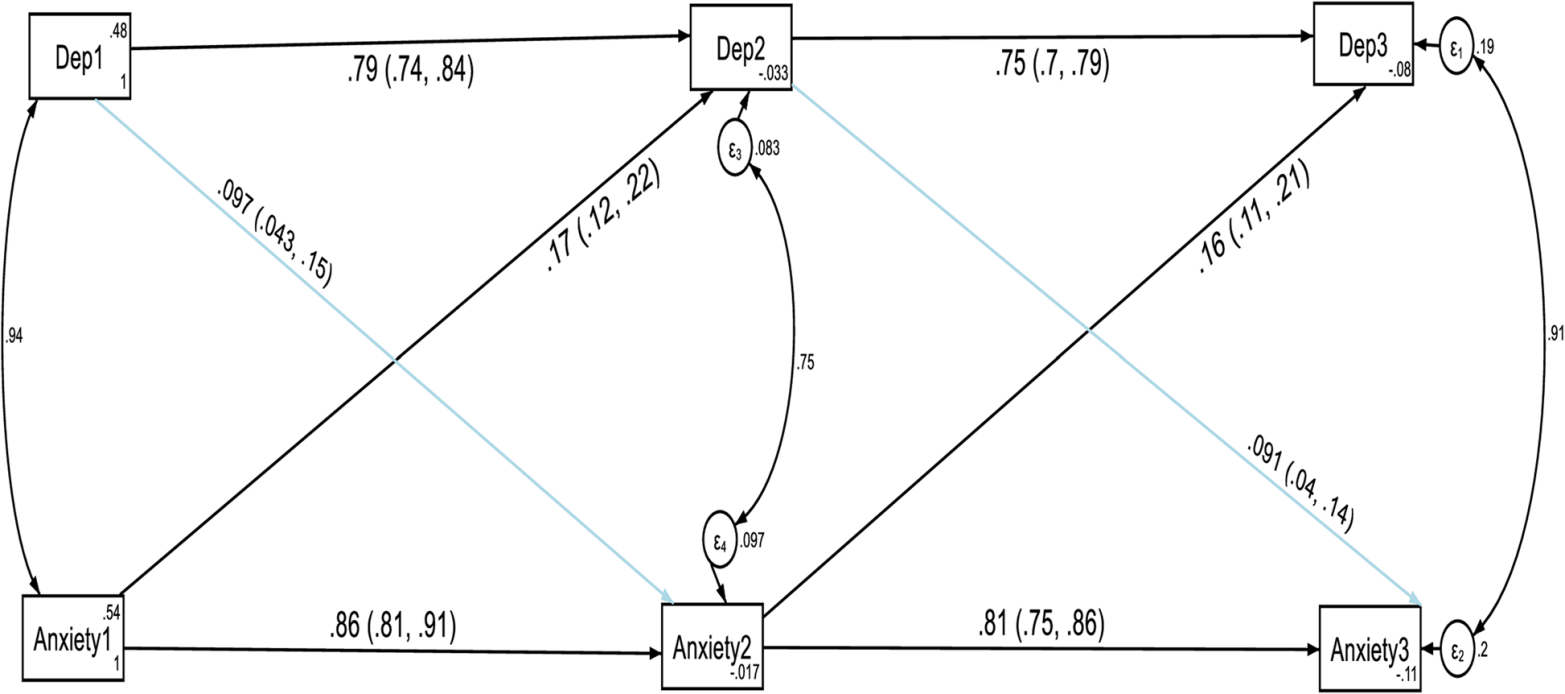
Cross-lagged autoregressive model assessing longitudinal stability and cross-lagged effects between depression and anxiety without controlling the direct and indirect effects of confounding variables. *Note:* All β’s are standardized estimates with their 95% CI

Also, the constrained model for depression and anxiety where the autoregressive and cross-lagged path coefficients were constrained to be equal across three time points did not significantly differ from the unconstrained model where the parameters were freely estimated as evidenced by the fit indices test of difference (ΔCFI = 0.005, ΔTLI = 0.003, ΔRMSEA = 0.015). Model fit for the unconstrained model: CFI = 0.997, TLI = 0.990, SRMR = 0.003 and RMSEA = 0.085. Model fit for the constrained model: CFI = 0.992, TLI = 0.987, SRMR = 0.047 and RMSEA = 0.10. Thus, these results indicated that anxiety and depression showed factorial invariance across the three waves and we used the constrained model as the final approved model of this study. The results of the structural model are given in Fig. 4 and Table 4.

The autoregressive path coefficients revealed that anxiety at T1 and depression at T1 predicted anxiety at T2 and depression at T2 respectively without controlling the direct and indirect effects of confounding variables. In addition, anxiety at T2 and depression at T2 predicted anxiety at T3 and depression at T3 respectively without controlling the direct and indirect effects of confounding variables. The cross-lagged path coefficients indicated that there is a statistically significant cross-lagged reciprocal association between depression and anxiety symptoms from T1 to T2 and from T2 to T3 without controlling the direct and indirect effects of confounding variables. The prediction of depression at T2 by T1 anxiety (β = 0.17, p-value < 0.001), was stronger than the prediction of anxiety at T2 by T1 depression (β = 0.097, *p*-value < 0.001). Similarly, the prediction of depression at T3 by T2 anxiety (β = 0.16, *p*-value < 0.001), was stronger than the prediction of anxiety at T3 by T2 depression (β = 0.09, *p*-value < 0.001) without controlling the direct and indirect effects of confounding variables.

### The longitudinal direction of association between depression, anxiety and PTSD symptoms while controlling the direct and indirect effects of confounding variables

**Fig. 5:**
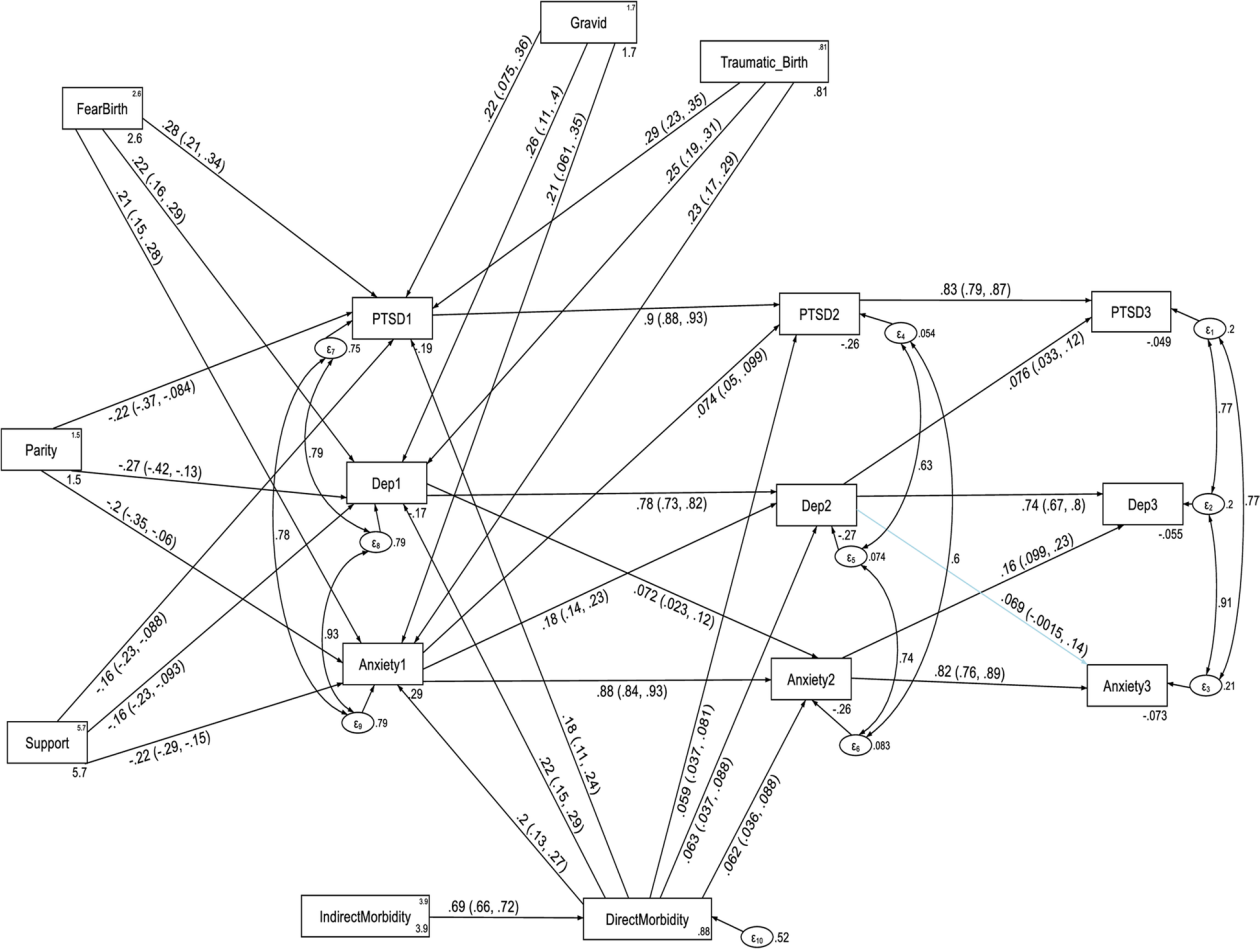
A modified cross-lagged autoregressive structural equation model assessing autoregressive and cross-lagged effects of depression, anxiety and PTSD symptoms while controlling the direct and indirect effects of confounding variables. *Note:* All β’s are standardized estimates with their 95% CI; only significant and marginally significant path coefficients are included in the path diagram

In order to examine the longitudinal relations between depression, anxiety and PTSD while controlling the direct and indirect effects of confounding variables, a cross-lagged autoregressive structural equation modeling was carried out. The stability and cross-lagged influence of PTSD, depression and anxiety symptoms while controlling the direct and indirect effects of confounding variables were presented in Fig. 5 and Table 5. The model fits the data well with CFI = 0.99, TLI = 0.98, SRMR = 0.035 and RMSEA = 0.058.

**Table 5:** Results of a modified cross-lagged autoregressive modelling between depression, anxiety and PTSD symptoms while controlling the direct and indirect effects of confounding variables among postpartum women in Northwest Ethiopia, 2021

Variable’s pathway	Type of mental health disorder at each follow up time								
	**T1 Depression**			**T2 Depression**			**T3 Depression**		
	**Direct effect**	**Indirect effect**	**Total effect**	**Direct effect**	**Indirect effect**	**Total effect**	**Direct effect**	**Indirect effect**	**Total effect**
	**β (95% CI)**	**β (95% CI)**	**β (95% CI)**	**β (95% CI)**	**β (95% CI)**	**β (95% CI)**	**β (95% CI)**	**β (95% CI)**	**β (95% CI)**
Direct morbidity Yes	3.11(2.09, 4.13) ^a^	No path	0.22(0.04) ^a^	0.83(0.57, 1.08) ^a^	2.72(1.82, 3.62) ^a^	3.55(2.61, 4.48) ^a^	No path	2.87(2.10, 3.63) ^a^	2.87(2.10, 3.63) ^a^
Fear of childbirth Yes	0.06(0.04, 0.07) ^a^	No path	0.22(0.03) ^a^	No path	0.05(0.04, 0.07) ^a^	0.05(0.04, 0.07) ^a^	No path	0.04(0.03, 0.05) ^a^	0.04(0.03, 0.05) ^a^
Gravidity	1.54(0.66, 2.41) ^a^	No path	0.26(0.07) ^a^	No path	1.31(0.55, 2.08) ^a^	1.31(0.55, 2.08) ^a^	No path	1.04(0.42, 1.67) ^a^	1.04(0.42, 1.67) ^a^
Parity	-1.68(-2.56, -0.79) ^a^	No path	-0.27(0.07) ^a^	No path	-1.415(-2.19, -0.64) ^a^	-1.415(-2.19, -0.64) ^a^	No path	-1.11(-1.74, -0.48) ^a^	-1.11(-1.74, -0.48) ^a^
Social support	-0.55(-0.79, -0.31) ^a^	No path	-0.16(0.04) ^a^	No path	-0.52(-0.73, -0.31) ^a^	-0.52(-0.73, -0.31) ^a^	No path	-0.44(-0.62, -0.27) ^a^	-0.44(-0.62, -0.27) ^a^
Traumatic birth Yes	3.46(2.57, 4.36) ^a^	No path	0.25(0.03) ^a^	No path	3.02(2.24, 3.80) ^a^	3.02(2.24, 3.80) ^a^	No path	.43(1.79, 3.07) ^a^	2.43(1.79, 3.07) ^a^
Indirect morbidity Yes	No path	0.15(0.39) ^a^	0.15(0.39) ^a^	No path	2.59(1.88, 3.30) ^a^	2.59(1.88, 3.30) ^a^	No path	2.09(1.52, 2.67) ^a^	2.09(1.52, 2.67) ^a^
T1 depression				0.72(0.68, 0.76) ^a^	No path	0.72(0.68, 0.76) ^a^	No path	0.49(0.44, 0.54) ^a^	0.49(0.44, 0.54) ^a^
T1 anxiety				0.17(0.13, 0.22) ^a^	No path	0.17(0.13, 0.22) ^a^	No path	0.24(0.19, 0.29) ^a^	0.24(0.19, 0.29) ^a^

^a^ p-value ≤ 0.001, ^b^ p-value < 0.01, β is unstandardized estimate

The autoregressive analyses revealed that depressive, anxiety and PTSD symptoms were stable over time which indicates that participants who suffered from depression, anxiety and PTSD in Time 1 tended to suffer from depression, anxiety and PTSD both in Time 2 and Time 3.

Depressive and anxiety symptoms have a statistically significant cross-lagged effect on PTSD symptoms but not vice versa. The cross-lagged analyses revealed that T1 anxiety symptoms predicted T2 PTSD symptoms and T2 depression symptoms predicted T3 PTSD symptoms. In other words, suffering from anxiety at time 1 increased the likelihood of PTSD at time 2 and suffering from depression at time 2 increased the likelihood of PTSD at time 3. However, suffering from PTSD did not change the likelihood of anxiety or depression in subsequent waves of measurement.

There was also a statistically significant cross-lagged association between depression and anxiety symptoms with vice versa at Time 1 but not at Time 2. Time 1 depression symptoms increased the likelihood of anxiety at time 2 but this pattern was not extended to time 3. In contrast, time 1 anxiety symptoms predicted the likelihood of time 2 depression and time 2 anxiety symptoms also predicted the likelihood of time 3 depression (see Table 5). Moreover, the prediction of Time 2 depression by Time 1 anxiety (β = 0.17, *p*-value < 0.001), was stronger than the prediction of Time 2 anxiety by Time 1 depression (β = 0.065, *p*-value = 0.004).

### Direct, indirect and total effects of variables associated with depression, anxiety and PTSD without controlling the autoregressive and cross-lagged effects at each follow up period.

A longitudinal path analyses were carried out using structural equation model to examine the direct and indirect association of variables with depression, anxiety and PTSD without controlling the autoregressive and cross-lagged effects at each follow up period. The structural equation model for PTSD fits the data well according to various fit indices (CFI = 0.969, TL = 0.879, RMSEA = 0.096 and SRMR = 0.033).

**Fig. 6:**
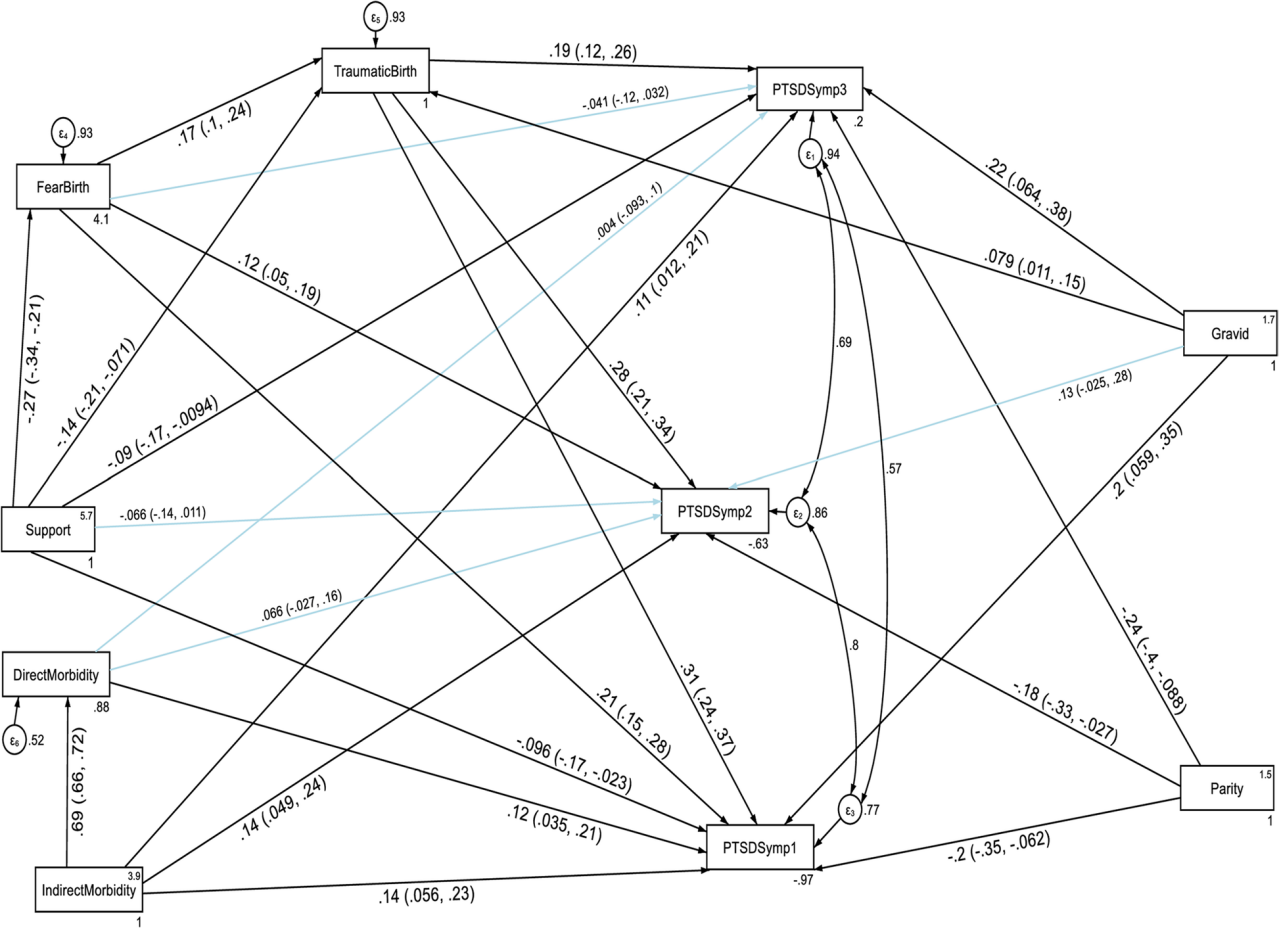
A modified structural equation model of the factors associated with PTSD symptoms without controlling the autoregressive and cross-lagged effects in postpartum women, Northwest Ethiopia. Note: β’s are standardized estimates with 95% CI

As shown in Fig. 6 and Table 6, direct and indirect maternal morbidity, fear of childbirth, multigravidity and perceived traumatic childbirth had a direct positive effect on PTSD at the first follow up period(T1). At the second follow up period (T2), indirect maternal morbidity, fear of birth and perceived traumatic birth had a direct positive effect on PTSD. At the third follow up period (T3), indirect maternal morbidity, perceived traumatic birth and multigravidity had also a direct positive effect on PTSD. Indirect maternal morbidity had both a direct positive effect and an indirect positive effect through direct maternal morbidity on PTSD score at T1. This indicates that PTSD score at the first follow up period was increased by 0.097 directly (β = 0.097, p-value = 0.002) for women who had indirect maternal morbidity compared to their counterpart and by 0.057 indirectly (β = 0.057, *p*-value = 0.007) through direct maternal morbidity (see Table 6 and Fig. 6).

**Table 6:** Direct, indirect and total effects of variables associated with anxiety and PTSD without controlling the autoregressive and cross-lagged effects, among postpartum women in Northwest Ethiopia, 2021

**Variable’s pathway**	**Type of mental health disorder at each follow up period**								
	**T1 Anxiety**			**T2 Anxiety**			**T3 Anxiety**		
	**Direct effect**	**Indirect effect**	**Total effect**	**Direct effect**	**Indirect effect**	**Total effect**	**Direct effect**	**Indirect effect**	**Total effect**
	**β (95% CI)**	**β (95% CI)**	**β (95% CI)**	**β (95% CI)**	**β (95% CI)**	**β (95% CI)**	**β (95% CI)**	**β (95% CI)**	**β (95% CI)**
Direct morbidity Yes	0.13(0.06, 0.20) ^a^	No path	0.13(0.06, 0.20) ^a^	0.13(0.06, 0.20) ^a^	No path	0.13(0.06, 0.20) ^a^	Not significant	No path	Not significant
Fear of childbirth Yes	0.01(0.004,0.01) ^a^	0.001(0.0003, 0.001) ^a^	0.01(0.004,0.006) ^b^	0.004(0.003, 0.005) ^a^	0.001(0.0002,0.001) ^a^	0.01(0.004, 0.01) ^a^	0.002(0.002, 0.003) ^a^	0.001(0.0002,0.001) ^b^	0.003(0.002, 0.004) ^b^
Gravidity	0.07(0.02, 0.12) ^b^	0.01(0.004, 0.01) ^a^	0.08(0.03, 0.13) ^b^	0.08(0.03, 0.12) ^a^	0.01(0.0004, 0.01) ^b^	0.08(0.04, 0.13) ^a^	0.09(0.05, 0.13) ^a^	0.01(0.0004, 0.01) ^b^	0.09(0.05, 0.13) ^a^
Parity	-0.07(-0.12, -0.02) ^b^	No path	-0.07(-0.12, -0.02) ^b^	-0.08(-0.12, -0.03) ^a^	No path	-0.08(-0.12, -0.03) ^a^	-0.08(-0.12, -0.04) ^a^	No path	-0.08(-0.12, -0.04) ^a^
Social support	-0.04(-0.06, -0.03) ^a^	-0.02(-0.03, -0.02) ^a^	-0.07(-0.08, -0.05) ^a^	-0.04(-0.05, -0.02) ^a^	-0.02(-0.03, -0.02) ^a^	-0.06(-0.07, -0.05) ^a^	-0.02(-0.03, -0.01) ^a^	-0.01(-0.02, -0.01) ^a^	-0.03(-0.05, -0.02) ^a^
Traumatic birth Yes	0.16(0.11, 0.21) ^a^	No path	0.16(0.11, 0.21) ^a^	0.14(0.10, 0.20) ^a^	No path	0.14(0.10, 0.20) ^a^	0.13(0.09, 0.17) ^a^	No path	0.13(0.09, 0.17) ^a^
Indirect morbidity Yes	0.14(0.06, 0.22) ^a^	0.09(0.04, 0.15) ^a^	0.23(0.17, 0.30) ^a^	0.20(0.13, 0.27) ^a^	0.09(0.05, 0.14) ^a^	0.29(0.23, 0.35) ^a^	0.13(0.07, 0.20) ^a^	Not significant	0.17(0.12, 0.22) ^a^
**Variable’s pathway**	**Type of mental health disorder at each follow up period**								
	**T1 depression**			**T2 depression**			**T3 depression**		
	**Direct effect**	**Indirect effect**	**Total effect**	**Direct effect**	**Indirect effect**	**Total effect**	**Direct effect**	**Indirect effect**	**Total effect**
	**β (95% CI)**	**β (95% CI)**	**β (95% CI)**	**β (95% CI)**	**β (95% CI)**	**β (95% CI)**	**β (95% CI)**	**β (95% CI)**	**β (95% CI)**
Direct morbidity Yes	0.12(0.05, 0.19) ^a^	No path	0.12(0.05, 0.19) ^a^	0.13(0.06, 0.19) ^a^	No path	0.13(0.06, 0.19) ^a^	0.07(0.02, 0.13) ^b^	No path	0.07(0.02, 0.13) ^b^
Fear of childbirth Yes	0.004(0.003, 0.005) ^a^	0.001(0.0003,0.001) ^a^	0.01(0.004, 0.01) ^b^	0.003(0.002,0.004) ^a^	0.001(0.0002,0.001) ^a^	0.004(0.003,0.005) ^a^	0.002(0.001, 0.003) ^a^	0.001(0.0002,0.001) ^b^	0.002(0.002,0.003) ^b^
Gravidity	0.06(0.02, 0.11) ^b^	0.01(0.001, 0.01) ^b^	0.07(0.02, 0.12) ^b^	0.07(0.03, 0.12) ^a^	0.01(0.001, 0.01) ^b^	0.08(0.03, 0.12) ^a^	0.08(0.04, 0.12) ^a^	0.01(0.005, 0.009) ^b^	0.09(0.05, 0.12) ^a^
Parity	-0.07(-0.12, -0.03) ^b^	No path	-0.07(-0.12, -0.03) ^b^	-0.09(-0.13, -0.04) ^a^	No path	-0.09(-0.13, -0.04) ^a^	-0.08(-0.12, -0.04) ^a^	No path	-0.08(-0.12, -0.04) ^a^
Social support	-0.03(-0.05, -0.02) ^a^	-0.02(-0.03, -0.02) ^a^	-0.06(-0.07, -0.04) ^a^	-0.03(-0.04, -0.02) ^a^	-0.02(-0.02, -0.01) ^a^	-0.05(-0.06, -0.03) ^a^	-0.02(-0.03, -0.01) ^b^	- 0.01(-0.02, -0.01) ^a^	-0.03(-0.04, -0.02) ^a^
Traumatic birth Yes	0.18(0.13, 0.23) ^a^	No path	0.18(0.13, 0.23) ^a^	0.16(0.11, 0.20) ^a^	No path	0.16(0.11, 0.20) ^a^	0.14(0.10, 0.18) ^a^	No path	0.14(0.10, 0.18) ^a^
Indirect morbidity Yes	0.14(0.06, 0.21) ^a^	0.09(0.04, 0.14) ^a^	0.22(0.16, 0.28) ^a^	0.15(0.08, 0.22) ^a^	0.09(0.05, 0.14) ^a^	0.24(0.19, 0.30) ^a^	0.10(0.04, 0.16) ^a^	0.05(0.01, 0.09) ^b^	0.15(0.10, 0.20) ^a^
**Variable’s pathway**	**Type of mental health disorder at each follow up period**								
	**T1 PTSD**			**T2 PTSD**			**T3 PTSD**		
	**Direct effect**	**Indirect effect**	**Total effect**	**Direct effect**	**Indirect effect**	**Total effect**	**Direct effect**	**Indirect effect**	**Total effect**
	**β (95% CI)**	**β (95% CI)**	**β (95% CI)**	**β (95% CI)**	**β (95% CI)**	**β (95% CI)**	**β (95% CI)**	**β (95% CI)**	**β (95% CI)**
Direct morbidity Yes	0.08 (0.02, 0.14) ^b^	No path	0.08 (0.02, 0.14) ^b^	Not significant	No path	Not significant	Not significant	No path	Not significant
Fear of childbirth Yes	0.01(0.001, 0.003) ^a^	0.001(0.0003, 0.001) ^a^	0.003(0.002,0.004) ^a^	0.001(0.001, 0.002) ^a^	0.001(0.001,0.002) ^a^	0.002(0.001,0.002) ^a^	Not significant	0.001(0.001,0.002) ^a^	Not significant
Gravidity	0.05(0.02, 0.09) ^b^	0.007(0.001, 0.012) ^b^	0.06(0.02, 0.10) ^b^	Not significant	0.005(0.001, 0.009) ^b^	Not significant	0.04(0.01, 0.06) ^b^	0.003(0.002,.005) ^b^	0.04(0.01, 0.06) ^b^
Parity	-0.06(-0.10, -0.02) ^b^	No path	-0.06(-0.01, -0.02) ^b^	-0.04(-0.08, -0.01) ^b^	No path	-0.04(-0.08, -0.01) ^b^	-0.04(-0.07, -0.01) ^b^	No path	-0.04(-0.07, -0.01) ^b^
Social support	-0.02(-0.03, -0.01) ^b^	-0.02(-0.02, -0.01) ^a^	-0.03(-0.04, -0.02) ^a^	Not significant	-0.01(-0.02, -0.01) ^a^	-0.02(-0.03, -0.01) ^a^	-0.01(-0.02, -0.001) ^b^	Not significant	-0.01(-0.02, -0.003) ^b^
Traumatic birth Yes	0.19(0.15, 0.23) ^a^	No path	0.19(0.15, 0.23) ^a^	0.14(0.11, 0.18) ^a^	No path	0.14(0.11, 0.18) ^a^	0.07(0.05, 0.10) ^a^	No path	0.07(0.05, 0.10) ^a^
Indirect morbidity Yes	0.10(0.04, 0.16) ^b^	0.06(0.02, 0.10) ^b^	0.15(0.11, 0.20) ^a^	0.08(0.03, 0.14) ^b^	Not significant	0.11(0.06, 0.15) ^a^	0.05(0.01, 0.09) ^b^	Not significant	0.05(0.01, 0.08) ^b^

^a^ p-value ≤ 0.001, ^b^ p-value < 0.01, β is unstandardized estimate

In contrast, multiparity had a direct negative effect on PTSD at the first(T1), second(T2) and third(T3) follow up period. Similarly, higher social support had a direct negative effect on PTSD at the first and third follow up period. As social support increased by one unit score, the mother’s estimated PTSD score at T1 decreased by 0.096 (β = -0.096, *p*-value = 0.01). With a one unit increase in social support score, the PTSD score at T3 also decreases by 0.09 (β = -0.09, *p*-value = 0.03) see Fig. 6.

The structural equation model for anxiety also fitted the data well according to various fit indices (CFI = 0.975, TL = 0.901, RMSEA = 0.096 and SRMR = 0.039). With regard to anxiety, indirect maternal morbidity, fear of childbirth, multigravidity and perceived traumatic childbirth had a direct positive effect on anxiety at the first (T1), second (T2) and third (T3) follow up period. Direct maternal morbidity had also a direct positive effect on anxiety symptoms at the first (T1) and second (T2) follow up period.

Compared to their counterparts, the anxiety score of women with indirect maternal morbidity at the first (T1) and second (T2) follow up period was increased by 0.094 (β = 0.094, *p*-value 0.027 and 0.025 respectively) indirectly through direct maternal morbidity see Table 6.

**Fig. 7:**
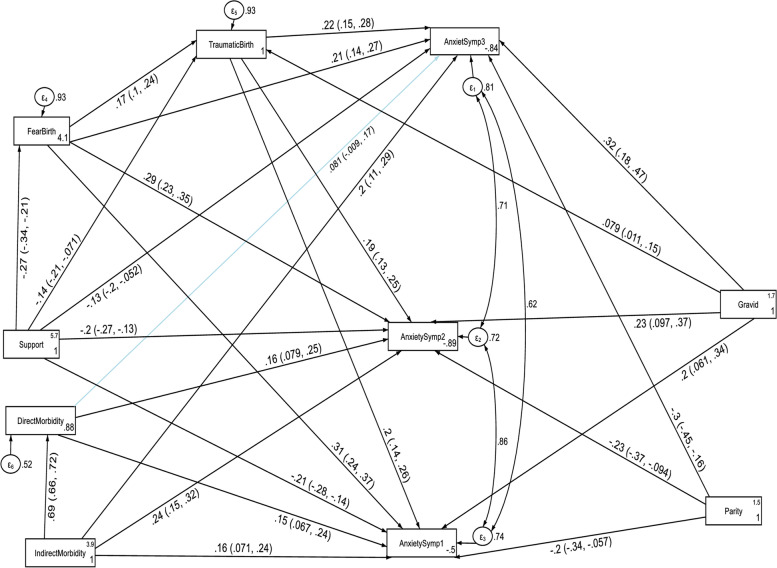
A modified structural equation model of the factors associated with anxiety symptoms without controlling the autoregressive and cross-lagged effects in postpartum women, Northwest Ethiopia. Note: β’s are standardized estimates with 95% CI

In contrast, multiparity had a direct negative effect on anxiety at the first(T1), second(T2) and third(T3) follow up period. Similarly, higher social support had both a direct and indirect negative effect on anxiety at the first and third follow up period. As social support increased by one unit score, the mother’s estimated anxiety score at T1, T2 and T3 decreased by 0.042 (β = -0.042, *p*-value < 0.001), 0.038 (β = -0.038, *p*-value < 0.001) and 0.02 (β = -0.019, *p*-value < 0.001) respectively. In addition, with a one unit increase in social support score, the anxiety score at T1, T2 and T3 decreased by 0.024 (β = -0.024, *p*-value < 0.001), 0.021 (β = -0.021, *p*-value < 0.001) and 0.015 (β = -0.015, *p*-value < 0.001) respectively, indirectly through perceived traumatic birth (see Fig. 7 and Table 6).

**Fig. 8:**
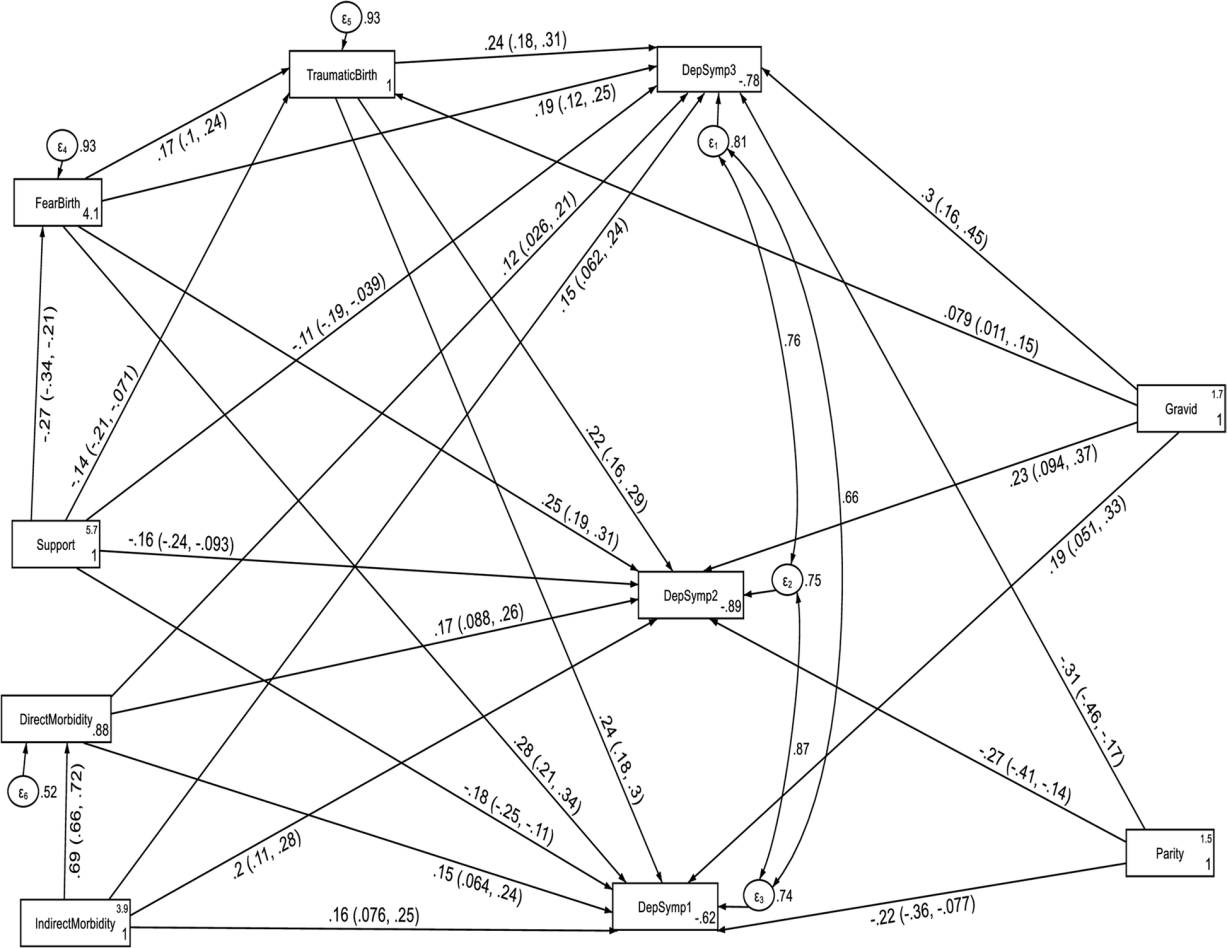
A modified structural equation model of the factors associated with anxiety symptoms without controlling the autoregressive and cross-lagged effects in postpartum women, Northwest Ethiopia. Note: β’s are standardized estimates with 95% CI

The structural equation model for depression fitted the data well according to various fit indices (CFI = 0.976, TL = 0.906, RMSEA = 0.096 and SRMR = 0.038). As shown in Fig. 8 and Table 6, direct and indirect maternal morbidity, fear of childbirth, multigravidity and perceived traumatic childbirth had a direct positive effect on depression throughout follow up period (T1, T2 and T3). Indirect maternal morbidity had also an indirect positive effect through direct maternal morbidity on depression score at T1, T2 and T3. In addition, multigravidity had an indirect positive effect through perceived traumatic birth on depression score at T1, T2 and T3. In contrast, multiparity had a direct negative effect on depression at the first(T1), second(T2) and third(T3) follow up period. Similarly, higher social support had a direct negative effect on depression and an indirect negative effect through fear of childbirth and perceived traumatic birth throughout the follow up period.

### Direct, indirect and total effects of variables associated with anxiety and PTSD symptoms while controlling the autoregressive and cross-lagged effects

As shown in Fig. 5 and Table 7, direct maternal morbidity, fear of childbirth, gravidity and perceived traumatic childbirth had a direct positive effect on Time 1 anxiety. Indirect maternal morbidity had also an indirect positive effect on Time 1 anxiety. In contrast, parity and social support had a direct negative effect on Time 1 anxiety. As social support increased by one standard deviation (from low to high), the mother’s estimated Time 1 anxiety score decreased by 0.22 standard deviations (total standardized effect = -0.22). Direct maternal morbidity had both a direct (unstandardized β = 0.81) and an indirect (unstandardized β = 2.50) positive effect on Time 2 anxiety score; meaning anxiety score was increased by 3.31 standard deviation (total unstandardized effect = 3.31) for participants who had direct maternal morbidity compared to those without direct maternal morbidity (see Table 7 and Fig. 5).

**Table 7:** Direct, Indirect and total effects of variables associated with anxiety and PTSD while controlling the autoregressive and cross-lagged effects, among postpartum women in Northwest Ethiopia, 2021

**Variable’s pathway**	**Type of mental health disorder at each follow up time**								
	**T1 Anxiety**			**T2 Anxiety**			**T3 Anxiety**		
	**Direct effect**	**Indirect effect**	**Total effect**	**Direct effect**	**Indirect effect**	**Total effect**	**Direct effect**	**Indirect effect**	**Total effect**
	**β (95% CI)**	**β (95% CI)**	**β (95% CI)**	**β (95% CI)**	**β (95% CI)**	**β (95% CI)**	**β (95% CI)**	**β (95% CI)**	**β (95% CI)**
Direct morbidity Yes	2.80(1.80, 3.80) ^a^	No path	0.20(0.04) ^a^	0.81(0.54,1.07) ^a^	2.50(1.62, 3.38) ^a^	3.31(2.39, 4.23) ^a^	No path	2.61(1.88, 3.34) ^a^	2.61(1.88, 3.34) ^a^
Fear of childbirth Yes	0.05(0.04, 0.07) ^a^	No path	0.21(0.03) ^a^	No path	0.05(0.03, 0.06) ^a^	0.05(0.03, 0.06) ^a^	No path	0.04(0.03, 0.05) ^a^	0.04(0.03, 0.05) ^a^
Gravidity	1.21(0.35, 2.06) ^b^	No path	0.21(0.07) ^b^	No path	1.09 (0.34, 1.84) ^b^	1.09 (0.34, 1.84) ^b^	No path	0.87(0.28, 1.46) ^b^	0.87(0.28, 1.46) ^b^
Parity	-1.22(-2.08, -0.35) ^b^	No path	-0.20(0.07) ^b^	No path	-1.11 (-1.87, -0.34) ^b^	-1.11 (-1.87, -0.34) ^b^	No path	-0.89 (-1.49, -0.29) ^b^	-0.89 (-1.49, -0.29) ^b^
Social support	-0.72(-0.97, -0.48) ^a^	No path	-0.22(0.04) ^a^	No path	-0.63 (-0.84, -0.421) ^a^	-0.63 (-0.84, -0.421) ^a^	No path	-0.49 (-0.65, -0.32) ^a^	-0.49 (-0.65, -0.32) ^a^
Traumatic birth Yes	3.06(2.19, 3.93) ^a^	No path	0.23(0.03) ^a^	No path	2.74(1.97, 3.51) ^a^	2.74(1.97, 3.51) ^a^	No path	2.17(1.56, 2.77) ^a^	2.17(1.56, 2.77) ^a^
Indirect morbidity Yes	No path	0.14(0.38) ^a^	0.14(0.38) ^a^	No path	2.41 (1.72, 3.11) ^a^	2.41 (1.72, 3.11) ^a^	No path	1.90(1.36, 2.45) ^a^	1.90(1.36, 2.45) ^a^
T1 depression				0.06(0.02, 0.11) ^b^	No path	0.06(0.02, 0.11) ^b^	No path	0.09 (0.04, 0.14) ^b^	0.09 (0.04, 0.14) ^b^
T1 anxiety				0.82(0.77, 0.87) ^b^	No path	0.82(0.77, 0.87) ^b^	No path	0.61 (0.55, 0.66) ^a^	0.61 (0.55, 0.66) ^a^
**Variable’s pathway**	**Type of mental health disorder at each follow up time**								
	**T1 PTSD**			**T2 PTSD**			**T3 PTSD**		
	**Direct effect**	**Indirect effect**	**Total effect**	**Direct effect**	**Indirect effect**	**Total effect**	**Direct effect**	**Indirect effect**	**Total effect**
	**β (95% CI)**	**β (95% CI)**	**β (95% CI)**	**β (95% CI)**	**β (95% CI)**	**β (95% CI)**	**β (95% CI)**	**β (95% CI)**	**β (95% CI)**
Direct morbidity Yes	4.74(2.87, 6.61) ^a^	No path	4.74(2.87, 6.61) ^a^	1.42(1.02, 1.81) ^a^	4.22(2.60, 5.85) ^a^	5.64(3.97, 7.31) ^a^	No path	4.59 (3.25, 5.93) ^a^	4.59 (3.25, 5.93) ^a^
Fear of childbirth Yes	0.13(0.10, 0.17) ^a^	No path	0.13(0.10, 0.17) ^a^	No path	0.12(0.09, 0.14) ^a^	0.12(0.09, 0.14) ^a^	No path	0.09 (0.07, 0.11) ^a^	0.09 (0.07, 0.11) ^a^
Gravidity	2.45(0.85,4.04) ^a^	No path	2.45(0.85,4.04) ^a^	No path	2.15(0.76, 3.54) ^b^	2.15(0.76, 3.54) ^b^	No path	1.74 (0.64, 2.85) ^b^	1.74 (0.64, 2.85) ^b^
Parity	-2.57(-4.19, -0.96) ^b^	No path	-2.57(-4.19, -0.96) ^b^	No path	-2.25(-3.66, -0.85) ^b^	-2.25(-3.66, -0.85) ^b^	No path	-1.83 (-2.95, -0.72) ^b^	-1.83 (-2.95, -0.72) ^b^
Social support	-0.99(-0.14, -0.55) ^b^	No path	-0.99(-0.14, -0.55) ^b^	No path	-0.90(-1.29, -0.52) ^a^	-0.90(-1.29, -0.52) ^a^	No path	-0.73 (-1.04, -0.42) ^a^	-0.73 (-1.04, -0.42) ^a^
Traumatic birth Yes	7.45(5.82, 9.08) ^a^	No path	7.45(5.82, 9.08) ^a^	No path	6.46(5.04, 7.88) ^a^	6.46(5.04, 7.88) ^a^	No path	5.13(3.99, 6.27) ^a^	5.13(3.99, 6.27) ^a^
Indirect morbidity Yes	No path	3.46(2.07, 4.85) ^a^	3.46(2.07, 4.85) ^a^	No path	4.12(2.86, 5.37) ^a^	4.12(2.86, 5.37) ^a^	No path	3.35 (2.34, 4.36) ^a^	3.35 (2.34, 4.36) ^a^
T1 PTSD				0.81(0.79, 0.83) ^a^	No path	0.81(0.79, 0.83) ^a^	No path	0.60(0.57, 0.63) ^a^	0.60(0.57, 0.63) ^a^
T1 depression				0.13(0.09, 0.17) ^a^	No path	0.13(0.09, 0.17) ^a^	No path	0.09 (0.04, 0.14) ^b^	0.09 (0.04, 0.14) ^b^
T1 anxiety				No path	No path	No path	No path	0.12 (0.08, 0.15) ^a^	0.12 (0.08, 0.15) ^a^

^a^ p-value ≤ 0.001, ^b^ p-value < 0.01, β is unstandardized estimate

### Direct, indirect and total effects of variables associated with depression while controlling the autoregressive and cross-lagged effects

As can be seen in Fig. 5 and Table 8, direct maternal morbidity, fear of childbirth, gravidity, and perceived traumatic childbirth had a direct positive effect on Time 1 depression. Direct maternal morbidity had a direct positive effect (standardized β = 0.22) on Time 1 depression score, with an increased depression score of 0.22 standard deviations above those women who had no direct maternal morbidity (see Fig. 5). Parity and social support had a direct negative effect on Time 1 depression and an indirect negative effect on Time 2 and Time 3 depression scores.

**Table 8:** Direct, Indirect and total effects of variables associated with depression while controlling the autoregressive and cross-lagged effects, among postpartum women in Northwest Ethiopia, 2021

Variable’s pathway	Type of mental health disorder at each follow up time								
	**T1 Depression**			**T2 Depression**			**T3 Depression**		
	**Direct effect**	**Indirect effect**	**Total effect**	**Direct effect**	**Indirect effect**	**Total effect**	**Direct effect**	**Indirect effect**	**Total effect**
	**β (95% CI)**	**β (95% CI)**	**β (95% CI)**	**β (95% CI)**	**β (95% CI)**	**β (95% CI)**	**β (95% CI)**	**β (95% CI)**	**β (95% CI)**
Direct morbidity Yes	3.11(2.09, 4.13) ^a^	No path	0.22(0.04) ^a^	0.83(0.57, 1.08) ^a^	2.72(1.82, 3.62) ^a^	3.55(2.61, 4.48) ^a^	No path	2.87(2.10, 3.63) ^a^	2.87(2.10, 3.63) ^a^
Fear of childbirth Yes	0.06(0.04, 0.07) ^a^	No path	0.22(0.03) ^a^	No path	0.05(0.04, 0.07) ^a^	0.05(0.04, 0.07) ^a^	No path	0.04(0.03, 0.05) ^a^	0.04(0.03, 0.05) ^a^
Gravidity	1.54(0.66, 2.41) ^a^	No path	0.26(0.07) ^a^	No path	1.31(0.55, 2.08) ^a^	1.31(0.55, 2.08) ^a^	No path	1.04(0.42, 1.67) ^a^	1.04(0.42, 1.67) ^a^
Parity	-1.68(-2.56, -0.79) ^a^	No path	-0.27(0.07) ^a^	No path	-1.415(-2.19, -0.64) ^a^	-1.415(-2.19, -0.64) ^a^	No path	-1.11(-1.74, -0.48) ^a^	-1.11(-1.74, -0.48) ^a^
Social support	-0.55(-0.79, -0.31) ^a^	No path	-0.16(0.04) ^a^	No path	-0.52(-0.73, -0.31) ^a^	-0.52(-0.73, -0.31) ^a^	No path	-0.44(-0.62, -0.27) ^a^	-0.44(-0.62, -0.27) ^a^
Traumatic birth Yes	3.46(2.57, 4.36) ^a^	No path	0.25(0.03) ^a^	No path	3.02(2.24, 3.80) ^a^	3.02(2.24, 3.80) ^a^	No path	2.43(1.79, 3.07) ^a^	2.43(1.79, 3.07) ^a^
Indirect morbidity Yes	No path	0.15(0.39) ^a^	0.15(0.39) ^a^	No path	2.59(1.88, 3.30) ^a^	2.59(1.88, 3.30) ^a^	No path	2.09(1.52, 2.67) ^a^	2.09(1.52, 2.67) ^a^
T1 depression				0.72(0.68, 0.76) ^a^	No path	0.72(0.68, 0.76) ^a^	No path	0.49(0.44, 0.54) ^a^	0.49(0.44, 0.54) ^a^
T1 anxiety				0.17(0.13, 0.22) ^a^	No path	0.17(0.13, 0.22) ^a^	No path	0.24(0.19, 0.29) ^a^	0.24(0.19, 0.29) ^a^

^a^ p-value ≤ 0.001, ^b^ p-value < 0.01, β is unstandardized estimate

## Discussion

### Prevalence rates of depression, anxiety and PTSD

In this study, the prevalence rates of depression across the three follow up periods were 15.5%, 12.9% and 8.6% respectively. The prevalence of anxiety symptoms during the same follow up periods were 18.5%, 15.5% and 9.5% respectively which was more prevalent than the prevalence rates of depression. This supports the evidence that anxiety may be as or more prevalent than depression during the perinatal period [9, 47]. In one study, it has been reported that the prevalence of anxiety disorder during the early postpartum period is 17.1% exceeding that of depression 4.8% [9]. Similar finding has been also reported by another study in which postpartum anxiety was very common (17%) during the postpartum period and is far more prevalent than depression (6%) after childbirth [47]. Symptoms of anxiety and depression after childbirth might be due to the physical and emotional stress and lifestyle changes that occur after this major life event [47].

The rates of postnatal depression (15.5%) at the 6^th^ week, (12.9%) at the 12^th^ week and (8.6%) at the 18^th^ week of postpartum were consistent with previous studies conducted in rural Ethiopia which reported prevalence rates of 12.2% to 22.1% [48, 49]. However, the prevalence of postnatal depression in this study was lower than other studies conducted in other urban areas of Ethiopia which reported prevalence rates of 22.4% to 33.2% [50–52]and in Turkey which was 32.6% [53].

The difference might be due to cultural and socioeconomic differences between study areas and varying study methodologies with different assessment time points, sample sizes, and diagnostic criteria.

Importantly, among women with symptoms of anxiety and depression at the first follow up period (6^th^ week of postpartum), 15.5% and 9.5% of women had anxiety and 12.9% and 8.6% of them had depression at the second (12^th^ week of postpartum) and third (18^th^ week of postpartum) follow up time, confirming the persistence of postnatal anxiety and depression from early to late postpartum period [53]. The persistence of postpartum anxiety and depression might reflect difficulties in adjusting to physiological and psychological changes that occur after birth and this highlights the significance of routine screening of postnatal anxiety and depression.

With regard to postpartum PTSD, a prevalence rate of 9.7%, 6.8% and 3.5% was observed in our study at the 6^th^, 12^th^ and 18^th^ week of postpartum respectively. This is consistent with a study conducted among perinatal women in Turkey [53]. However, 9.7% and 6.8% prevalence of PTSD in our study, is considerably higher than the finding of a systematic review which reported 4% postpartum PTSD [20]. The postpartum PTSD symptoms might be due to difficult childbirth experiences triggering new onset of PTSD or exacerbating pre-existing PTSD symptoms. Other possible explanation could be, a difficult birth might retrigger PTSD which was experienced in early life. The prevalence of 3.5% PTSD at the 18^th^ week of postpartum, suggests that PTSD after childbirth might be chronic and indicates that some women with negative birth experiences maintained their negative perception of childbirth and their symptoms until 18^th^ week of postpartum.

### The cross-lagged autoregressive effects between depression, anxiety and PTSD

Measuring patterns of relations between PTSD, anxiety and depression over time revealed that anxiety symptoms at the first follow up period leads to PTSD symptoms at the second follow up period and depression symptoms at the second follow up period leads to PTSD at the third follow up period. This indicates that PTSD is secondary to depression and anxiety which implies that anxiety and depression are a casual risk factor for PTSD. This is in congruent with previous literatures which reported that symptoms of anxiety and depression lead to PTSD in line with the diathesis-stress model [13, 18, 26, 54]. However, other studies have reported an inverse direction of relationships that PTSD symptoms leads to depressive symptoms [55, 56]. Given these diverse results, it is important for future researchers to explore theoretically driven models to explain the directionality of the relationships between postpartum depression, anxiety and PTSD. The possible reason for which depressive symptoms leads to PTSD, might be due to the negative affect and anhedonia (lack of pleasure) by depressive symptoms, which in turn increases the risk for PTSD symptoms [26]. Other possible reason might be depressive symptoms' adverse impact on the self, which in turn increases the risk for PTSD as it has been evidenced that negative cognitions regarding the self were prospectively associated with an increase in PTSD symptoms after childbirth [26, 57]. Moreover, it might be also due to depressive symptoms' effect on impaired motivation for fear extinction [26].

The possible justification for which anxiety symptoms leads to PTSD might be due to childbirth related negative emotions which could overwhelm the mother and induce dissociative symptoms that interfere with the integration of traumatic memories [13]. Such emotional reactions likely indicate that birth was appraised as threatening and difficult to control which is confirmed by the result of this study that 39.5% of mothers perceived childbirth as traumatic.

In addition, anxiety symptoms at the first follow up period leads to depression symptoms at the second follow up period more than vice versa. Furthermore, anxiety symptoms at the second follow up period also leads to depression symptoms at the third follow up period. This also implies that anxiety is a causal risk factor for depression. Our findings are consistent with those of previous studies which indicated risk of postpartum depression to be associated with higher levels of self-reported anxiety symptoms [58–61].

### Direct, indirect and total effects of variables associated with depression, anxiety and PTSD

In this study, direct maternal morbidity, fear of childbirth, gravidity, parity, family size, social support, perceived traumatic childbirth and indirect maternal morbidity were found to have a direct and indirect association with depression, anxiety and PTSD.

Direct maternal morbidity has a direct and positive association with anxiety, depression and PTSD at the first and second follow up period. It has also an indirect positive association with these events at the second and third follow up period through their respective previous waves of measurement. Indirect maternal morbidity was also found to have a positive indirect association with all the above events through direct maternal morbidity and their respective previous waves of measurement. This is consistent with previous literatures which have shown that postpartum risks of onset of depression, post-traumatic stress and anxiety are higher among women whose pregnancy included obstetrical complications, compared with women with uneventful pregnancies [62–66].

Consistent with previous literatures [18, 63, 67, 68], fear of childbirth, gravidity and perceived traumatic childbirth were found to increase the risk of depression, anxiety and PTSD directly at the first follow up period and indirectly at the second and third follow up period. In contrast, multiparity decrease the risk of depression, anxiety and PTSD at the first follow up period and indirectly at the second and third follow up period. In addition, large family size is protective of anxiety, depression and PTSD directly at the first follow up period and indirectly at the second and third follow up period. Mixed findings were reported for parity including primiparity being a risk factor in one study [47] and multiparity in another study [10]. In a systematic review, it has been reported that nulliparity to be a predisposing factor for PTSD which is in line with our study finding [68]. Therefore, further research is needed to substantiate this.

Higher social support also decreases the risk of depression, anxiety and PTSD directly at the first follow up period and indirectly at the second and third follow up period which is in line with previous literatures [1, 2, 68, 69]. This might be due to the buffering effect of higher social support on negative cognitions since it provides needed social, emotional and physical provisions [1].

### Strength and limitation of the study

Strength of this study is the investigation of longitudinal associations among symptoms of depression, anxiety and PTSD using a cross-lagged autoregressive structural equation modeling. Our findings regarding the effect of depressive and anxiety symptoms on PTSD might highlight the centrality of depressive and anxiety symptoms as a prevalent and quite threatening experience felt by women during the postpartum period. However, this study was not without limitations. First, antenatal factors like depression and anxiety during pregnancy and prior PTSD which may influence rates of these events in the postpartum period were not included in the study. Thus, it would have been better to include antenatal depression, anxiety and prior PTSD as a confounding variable. Therefore, further studies are required to clarify this issue. Second, self-report questionnaires rather than clinical interviews were used to assess anxiety, depression and PTSD which might inflate prevalence rates. Although spuriousness was sometimes the limitation of cross-lagged structural equation modeling, we have collected longitudinal data on anxiety, depression and PTSD at the 6^th^, 12^th^ and 18^th^ week of postpartum period to determine the temporal sequencing of these disorders. Therefore, the issue of spurious findings during the evaluation of whether PTSD preceded depression and anxiety disorders or vice versa was controlled and minimized by doing so.

## Conclusion

The findings of this study showed that anxiety was more prevalent than depression and PTSD at the 6^th^, 12^th^ and 18^th^ week of postpartum period. With regard to the chronological relations among the three comorbid disorders, the finding of this study supports the diathesis-stress model that views anxiety and depression as a causal risk factor for PTSD. Anxiety was also found to be a causal risk factor for depression.

In the current study, direct maternal morbidity, fear of childbirth, higher gravidity, perceived traumatic childbirth and indirect maternal morbidity were found to have a direct and indirect positive association with depression, anxiety and PTSD. In addition, higher parity, higher family size and higher social support have a direct and indirect negative association with these mental health disorders.

Therefore, there should be effective postnatal screening to identify women with psychological problems. Early detection and treatment of anxiety and depression may reduce the likelihood of postnatal psychological disorders. Women with anxiety and depressive symptoms should also be screened for postpartum PTSD symptoms.

Women with direct and indirect maternal morbidities should be identified and treated early, in order to reduce the subsequent burden of anxiety, depression and PTSD. Adequate information about birth procedures and response to their needs should be given for women with fear of childbirth and perceived traumatic birth to decrease negative emotions of women and fear of childbirth. This would prevent the subsequent anxiety, depressive and PTSD symptoms. Interventions targeting to encourage social support may also help in increasing mothers’ coping and buffer negative cognitions so as to prevent symptoms of anxiety, depression and PTSD.

## Supplementary Information

**Additional file 1: S1.** Theoretical Model 1 for Modified Models in Figure 2, 3 and 4.

**Additional file 2: S2.** Theoretical Model 2 for Modified Model in Figure 5.

**Additional file 3: S3.** Theoretical Model 3 at Time 1 for Modified Models in Figure 6, 7 and 8.

**Additional file 4: S4.** Theoretical Model 4 at Time 2 for Modified Models in Figure 6, 7 and 8.

**Additional file 5: S5.** Theoretical Model 5 at Time 3 for Modified Models in Figure 6, 7 and 8.

**Additional file 6: S6.** Software functions for Figure 2 and Table 4.

**Additional file 7: S7.** Software functions for Figure 3 and Table 4.

**Additional file 8: S8.** Software functions for Figure 4 and Table 4.

**Additional file 9: S9.** Software functions for Figure 5, Tables 5, 7 and 8.

**Additional file 10: S10.** Software functions for Figure 6 and Table 6.

**Additional file 11: S11.** Software functions for Figure 7 and Table 6.

**Additional file 12: S12.** Software functions for Figure 8 and Table 6.

**Additional file 13: S13.** Software functions for the theoretical Model 1.

**Additional file 14: S14.** Software functions for the theoretical Model 2.

**Additional file 15: S15. **Software functions for the theoretical Model 3.

**Additional file 16: S16. **Software functions for the theoretical Model 4.

**Additional file 17: S17.** Software functions for the theoretical Model 5.

## Data Availability

Extra data is available from the corresponding author upon reasonable request.

## Abbreviations

ARCL: Autoregressive cross-lagged analysis
CFI: Comparative fit index
DASS: Depression, anxiety and stress scale
LMICs: Low- and middle-income countries
PCL-5: Posttraumatic Stress Disorder Checklist for DSM-5
RMSEA: Root-mean-square error of approximation
TES: Traumatic event scale
TLI: Tucker-Lewis Index
W-DEQ: Wijma Delivery Expectation/Experience Questionnaire
WHO: World Health Organization
PTSD: Posttraumatic stress disorder
