# Supplementary figures and images for: Longitudinal patterns of the relation between anxiety, depression and posttraumatic stress disorder among postpartum women with and without maternal morbidities in Northwest Ethiopia: a cross-lagged autoregressive structural equation modelling
Source: Arch Public Health. 2022 Oct 29;80:225. doi: 10.1186/s13690-022-00978-0 (PMC9617360; doi:10.1186/s13690-022-00978-0)

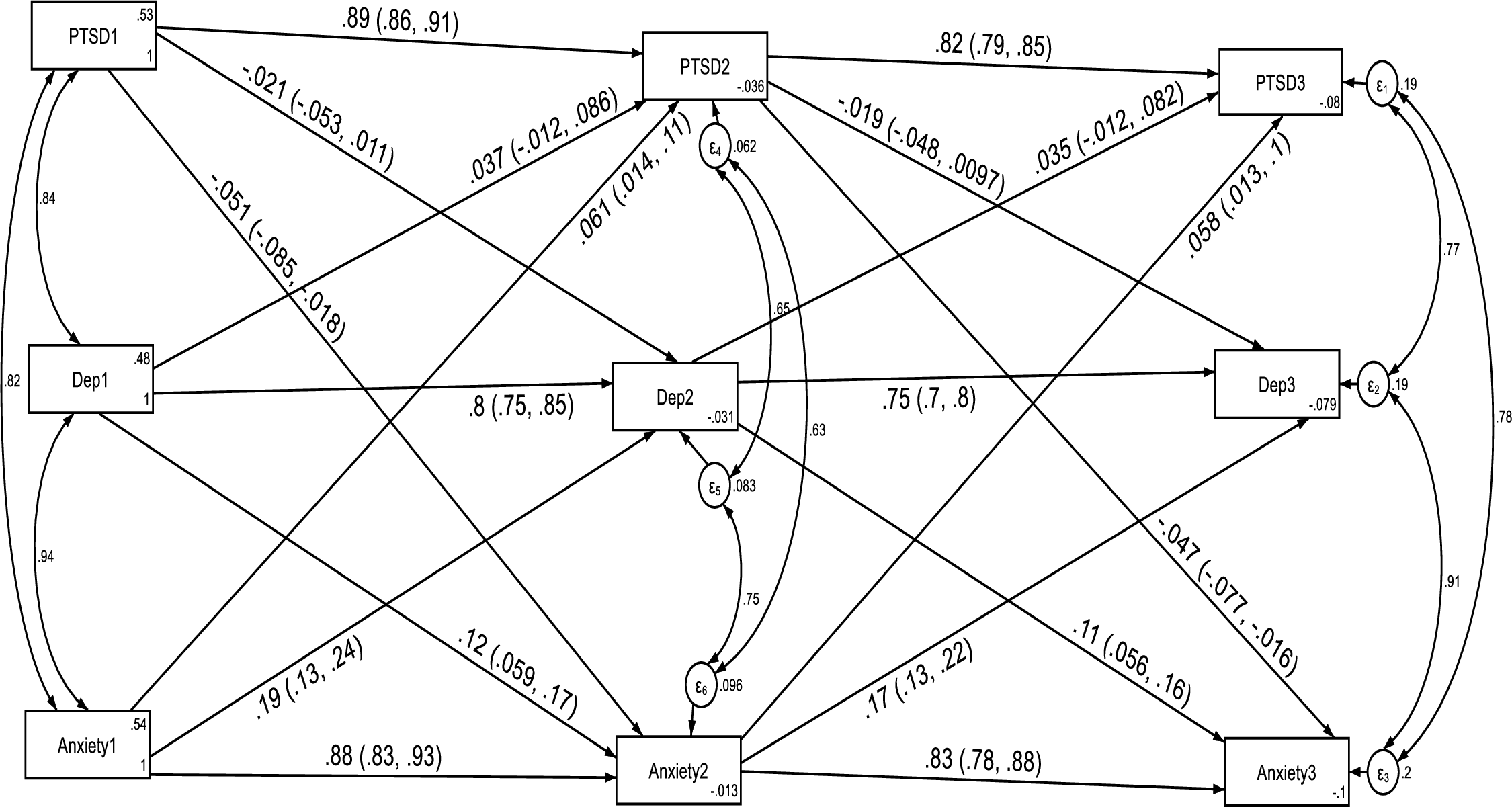

Supplement: Supplementary file 1 — Additional file 1: S1. Theoretical Model 1 for Modified Models in Figure 2, 3 and 4. [file 13690_2022_978_MOESM1_ESM.tif]

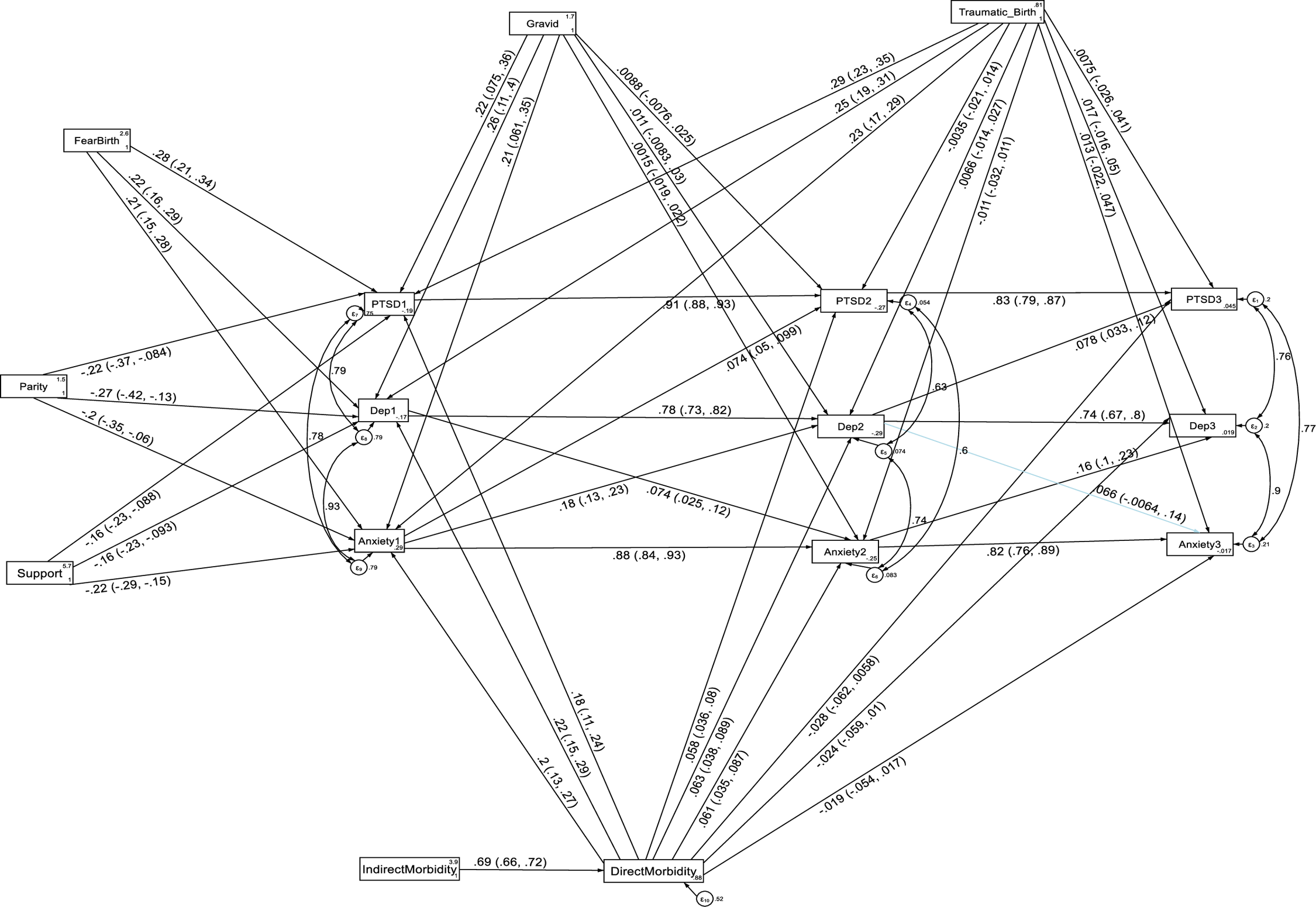

Supplement: Supplementary file 2 — Additional file 2: S2. Theoretical Model 2 for Modified Model in Figure 5. [file 13690_2022_978_MOESM2_ESM.tif]

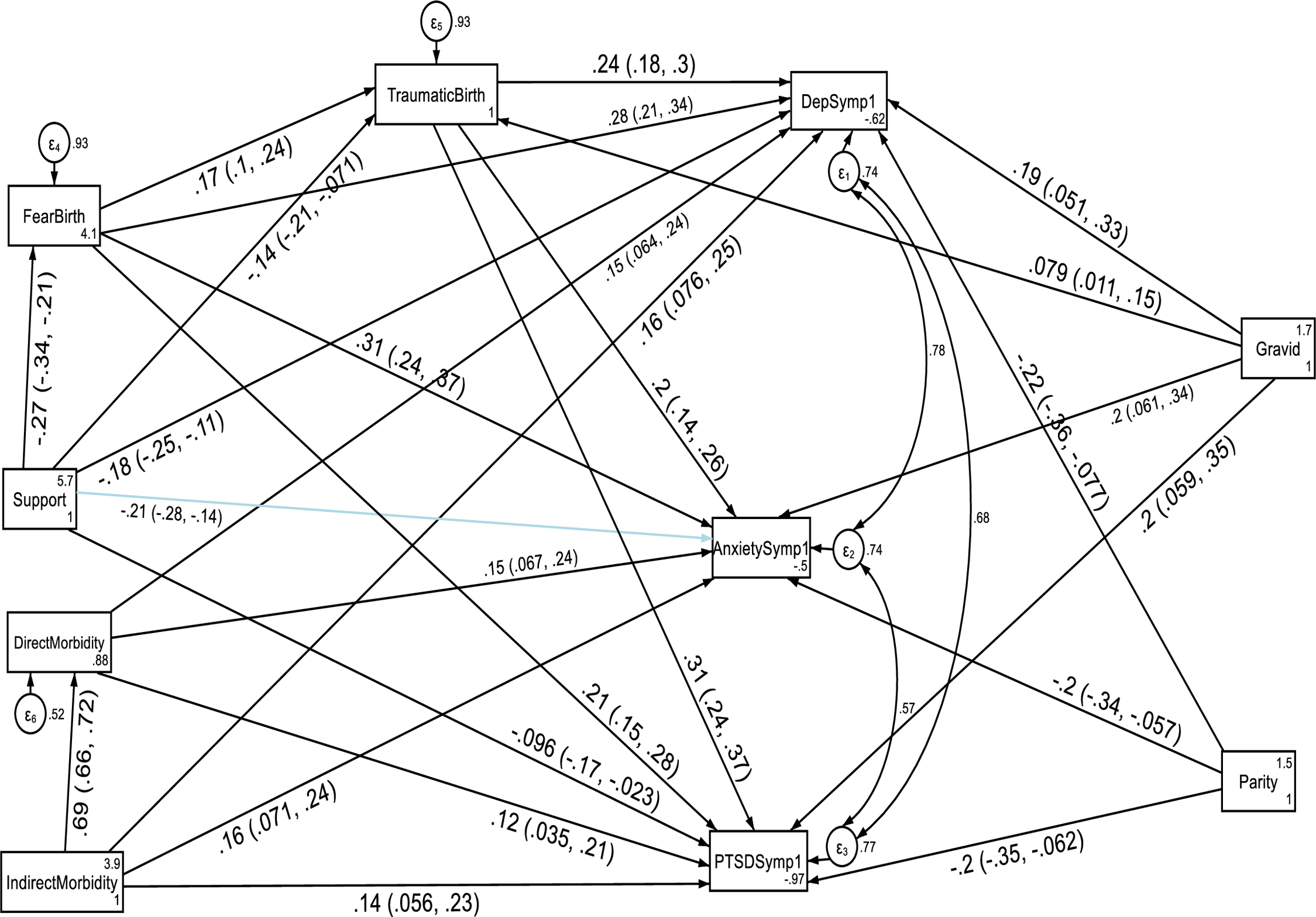

Supplement: Supplementary file 3 — Additional file 3: S3. Theoretical Model 3 at Time 1 for Modified Models in Figure 6, 7 and 8. [file 13690_2022_978_MOESM3_ESM.tif]

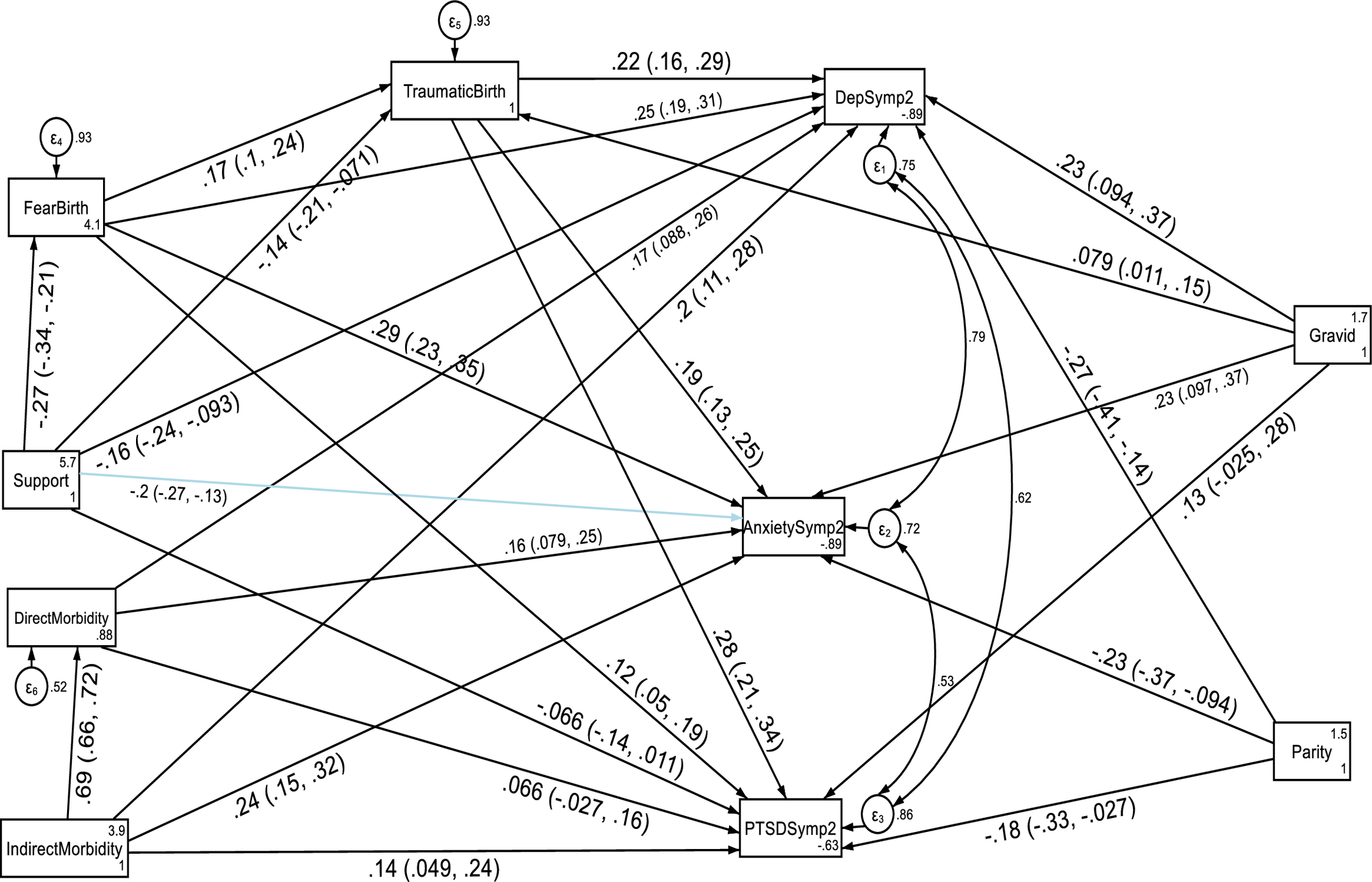

Supplement: Supplementary file 4 — Additional file 4: S4. Theoretical Model 4 at Time 2 for Modified Models in Figure 6, 7 and 8. [file 13690_2022_978_MOESM4_ESM.tif]

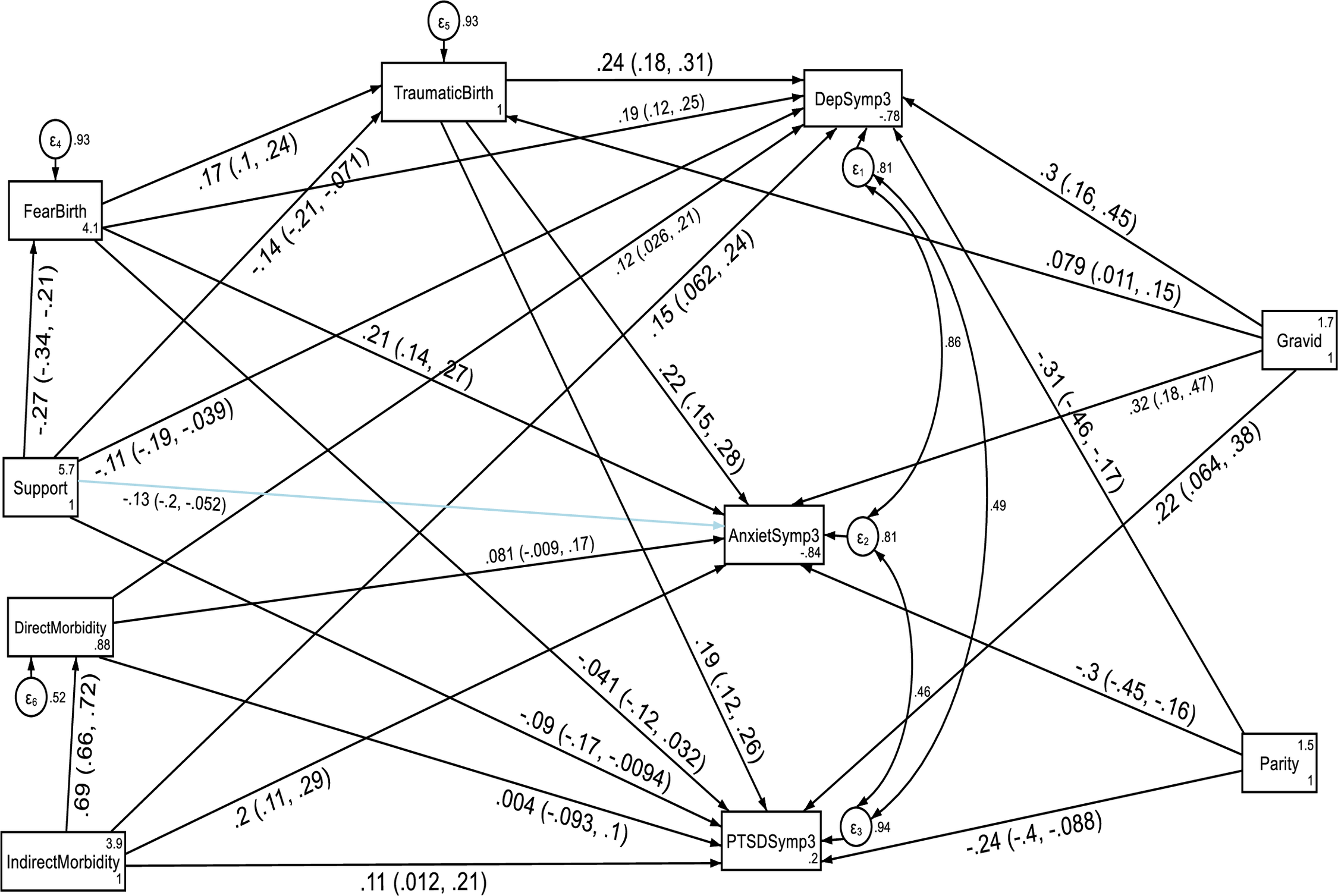

Supplement: Supplementary file 5 — Additional file 5: S5. Theoretical Model 5 at Time 3 for Modified Models in Figure 6, 7 and 8. [file 13690_2022_978_MOESM5_ESM.tif]
